# mRNA display reveals a class of high-affinity bromodomain-binding motifs that are not found in the human proteome

**DOI:** 10.1016/j.jbc.2023.105482

**Published:** 2023-11-20

**Authors:** Jason K.K. Low, Karishma Patel, Natasha Jones, Paul Solomon, Alexander Norman, Joshua W.C. Maxwell, Petr Pachl, Jacqueline M. Matthews, Richard J. Payne, Toby Passioura, Hiroaki Suga, Louise J. Walport, Joel P. Mackay

**Affiliations:** 1 School of Life and Environmental Sciences, University of Sydney, New South Wales, Australia; 2 School of Chemistry, University of Sydney, New South Wales, Australia; 3 Department of Chemistry, Graduate School of Science, The University of Tokyo, Bunkyo, Tokyo, Japan; 4 Protein-Protein Interaction Laboratory, The Francis Crick Institute, London, United Kingdom; 5 Department of Chemistry, Molecular Sciences Research Hub, Imperial College London, London, United Kingdom

**Keywords:** mRNA display, BET bromodomain, BRD3, structural biology

## Abstract

Bromodomains (BDs) regulate gene expression by recognizing protein motifs containing acetyllysine. Although originally characterized as histone-binding proteins, it has since become clear that these domains interact with other acetylated proteins, perhaps most prominently transcription factors. The likely transient nature and low stoichiometry of such modifications, however, has made it challenging to fully define the interactome of any given BD. To begin to address this knowledge gap in an unbiased manner, we carried out mRNA display screens against a BD—the N-terminal BD of BRD3—using peptide libraries that contained either one or two acetyllysine residues. We discovered peptides with very strong consensus sequences and with affinities that are significantly higher than typical BD–peptide interactions. X-ray crystal structures also revealed modes of binding that have not been seen with natural ligands. Intriguingly, however, our sequences are not found in the human proteome, perhaps suggesting that strong binders to BDs might have been selected against during evolution.

Biology is built on networks of protein–protein interactions that have affinities that span over 10 orders of magnitude (1). It has often been mooted that protein–protein interactions—particularly those involved in signaling networks—are not necessarily optimized for high affinity because lower affinities might provide higher sensitivity in response to environmental stimuli. For example, although SH3 domains often bind target polyproline motifs with affinities of 10 to 100 μM (2), *K*_*D*_s as low as 10 nM have been reported (3) demonstrating that the SH3–polyproline system is capable of affinities ∼1000-fold higher than the typical range.

Relatively weak interactions are abundant in transcriptional regulation, including in the recognition of histone post-translational modifications by “reader” domains. A prominent example is the interaction between bromodomains (BDs) and linear motifs containing acetyllysine (AcK) residues. BDs are small alpha-helical bundles found in ∼40 human proteins that mainly regulate gene expression (4). These domains harbor a deep pocket that specifically recognizes AcK-containing peptide motifs in both histones and other gene regulatory proteins such as transcription factors (5, 6, 7, 8). The affinities of these interactions lie in the range 10 to 200 μM (*e.g.*, (9, 10)), although we have recently shown that non-native cyclic peptides containing AcK residues can achieve nanomolar and even picomolar affinities (11), highlighting that higher affinities are, in principle, possible.

The BET family of BD-containing proteins (BRD2, BRD3, BRD4, and BRDT) each possess two BDs that can recognize motifs bearing either a single AcK residue or two AcK residues (di-AcK); di-AcK motifs generally have the consensus AcK-XX-AcK, where X is any amino acid (12, 13) (Fig. S1*B*). We and others have previously shown that transcription factors such as GATA1, Twist, E2F1, and MyoD1 contain diacetylated AcK-XX-AcK motifs that can be specifically recognized by *N*-terminal BDs from BET proteins (5, 6, 10). However, the large number of possible transcription factor acetylation events, as well as the likely nonstoichiometric and dynamic nature of these events, has hindered the creation of a comprehensive map of BD interactions (14).

In an effort to obtain an unbiased and broad-based view of BD target sequences, we used Random nonstandard Peptide Integrated Discovery (RaPID) mRNA display (15). Because we had previously shown that the *N*-terminal BD of BRD3 (BRD3–BD1) can bind several transcription factors (6), we created libraries of 9- or 12-residue peptides containing one or two AcK residues, respectively, and screened these libraries against BRD3–BD1 (Fig. S1*A*).

Peptides with very strong consensus sequences were isolated from the screens, and biophysical analysis of the most enriched peptide from the di-AcK screen revealed affinities for BET-family BDs of ∼1 to 2 μM, ∼5 times tighter than the strongest known single BD–substrate interaction (16). X-ray crystal structures of the mono-AcK and di-AcK peptides bound to BRD3–BD1 revealed the structural basis for these interactions, showing that they are unlike any interactions with known physiological ligands. Unexpectedly, these consensus sequences do not appear in the human proteome, suggesting that the tightest-binding target sequences for this domain have perhaps not been selected for during evolution. This finding is in line with the idea that the ability for interactions to be rapidly regulated is an important aspect of cellular processes such as gene regulation.

## Results

### RaPID screens against BRD3–BD1 select highly enriched AcK-containing sequences

**Figure 1:**
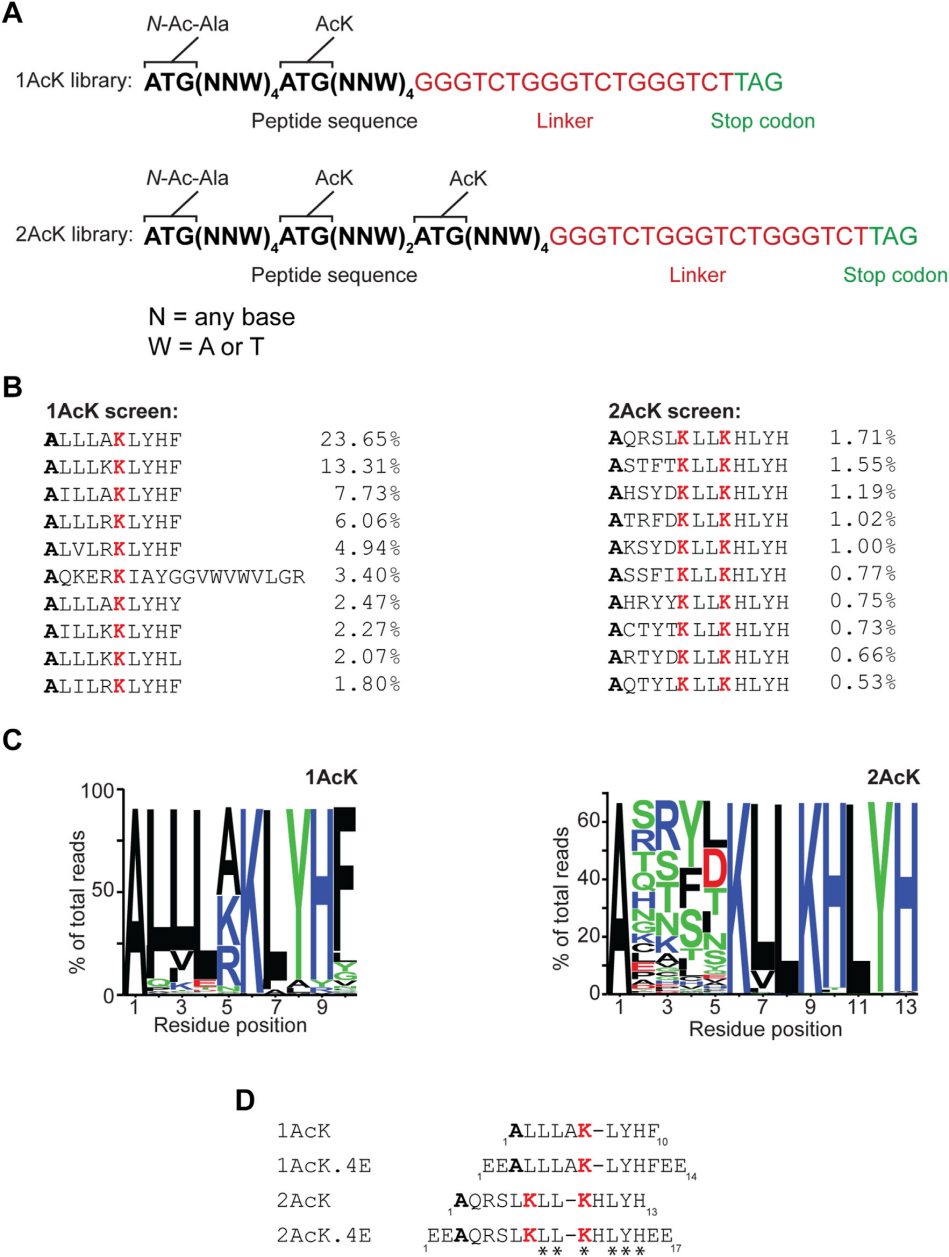
**RaPID screens for BRD3–BD1 binding peptides.***A*, library design for the 1AcK and 2AcK libraries. NNW codons encode any amino acid except Trp and Met. *B*, alignment of the 10 most highly represented sequences from each screen. The percentage of reads of each sequence is shown as a fraction of the total number of mapped reads in each screen. *Red* indicates acetyllysine, whereas *bold* indicates the N-terminal residue in the parent peptide that carries an acetyl group. The *top sequence* from each screen was synthesized for further analysis. *C*, sequence logo (generated from the 1000 most abundant sequences using WebLogo) showing the abundance of each amino acid in each position of the selected sequences. *D*, alignment of peptides selected and used in this study. Positions that are conserved across the sequences used are indicated with *asterisks* (∗) beneath the sequences. BD, bromodomain; RaPID, Random nonstandard Peptide Integrated Discovery.

To assess the substrate preferences for a BD in a comprehensive manner, we designed two DNA-encoded linear peptide libraries that used genetic code reprogramming to incorporate AcK at ATG codons (17) (Figs. 1*A* and S2). One library encoded peptides of the type ^*N*^AcA-X_4_-AcK-X_4_ (the 1AcK library), where X is any natural amino acid other than Trp and Met, and ^*N*^AcA is an N-terminally acetylated Ala. Given the ability of BET BDs to recognize sequences containing two AcK residues (most often separated by two residues), we also created a library with the composition ^*N*^AcA-X_4_-AcK-X_2_-AcK-X_4_ (the 2AcK library). We chose the first BD of the human BET-family protein BRD3 (BRD3–BD1) as a target and separately panned the two libraries against biotinylated BRD3–BD1 immobilized on streptavidin beads.

Following four rounds of mRNA display selection, the DNA libraries generated from each round were sequenced. Clear enrichment was observed even after only two rounds, and these preferred sequences were further enriched after four rounds (Table S1). Figure 1*B* shows the 10 most highly represented sequences from each selection. In line with the library design, each peptide contained either one or two AcK residues. Weighted consensus sequences derived from the top 1000 sequences (which comprised >80% and >60% of total DNA sequencing reads from the 1AcK and 2AcK selections, respectively) show the emergence of strong consensus sequences in the majority of the randomized positions (Fig. 1*C*). In addition, the most enriched sequence from each selection also conforms to this 1000-sequence consensus.

Substantial similarity was also observed between the sequences selected in the two experiments (Fig. 1*D*). Leu is highly enriched in the N-terminal part of each sequence and a Leu-Tyr-His sequence in the *C*-terminal part (Fig. 1*D*). Interestingly, although there appeared to be amino acid preferences in all positions in the single AcK library, almost no preference was observed in the positions *N* terminal to the first AcK in the 2AcK selection (Fig. 1, *B* and *C*).

### The most enriched 2AcK sequence binds BD1 domains with high affinity

**Figure 2:**
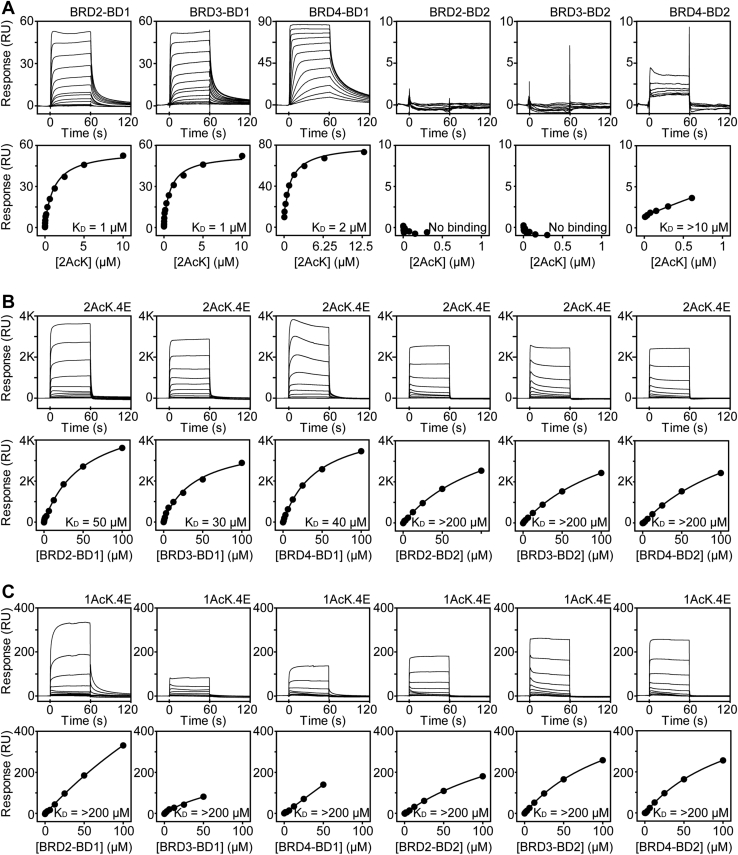
**SPR analysis ofBD–peptide interactions.***Top*, typical sensorgrams are shown for (*A*) one titration of each of the six indicated BDs with 2AcK, (*B*) titration of 2AcK.4E with each of the six BDs, and (*C*) titration of 1AcK.4E with each of the six BDs. *Bottom*, fits to a 1:1 binding model are shown; *K*_*D*_s are indicated on each plot. Each experiment was conducted at least three times, and geometric mean *K*_*D*_ values and associated uncertainties are given in Table 1. BD, bromodomain; SPR, surface plasmon resonance.

We synthesized the most enriched peptide from each selection (1AcK and 2AcK; Fig. 1, *B* and *D*) and used surface plasmon resonance (SPR) to assess their ability to bind each of the two BDs from the BET paralogs BRD2, BRD3, and BRD4, which were each immobilized on a streptavidin-coated SPR chip. The peptide 1AcK was not soluble at concentrations high enough to allow measurements to be made, given the weak affinity of the interaction. 2AcK was slightly more soluble and displayed robust and reproducible binding to each of the three BD1 domains; affinities of ∼1 to 2 μM to BRD3–BD1, BRD4–BD1, and BRD2–BD1 were measured using equilibrium steady state affinity analysis (Fig. 2*A* and Table 1); rate constants could not be measured because of the fast association and dissociation kinetics observed. Affinities for the BD2 domains were substantially lower with no measurable binding to BRD2–BD2 and BRD3–BD2, and a small degree of binding observed to BRD4–BD2, though an affinity could not be reliably determined.

**Table 1:** Dissociation constants for the peptides selected for study from RaPID selections against BRD3–BD1

Peptide	*Kᴅ* (μM)^a^					
	BRD2–BD1	BRD3–BD1	BRD4–BD1	BRD2–BD2	BRD3–BD2	BRD4–BD2
2AcK						
SPR	2	2	1	NB^b^	NB^b^	>10
ITC		10				
1AcK.4E						
SPR	>200	>200	>200	>200	>200	>200
NMR		ND^c^			40	
2AcK.4E						
SPR	70	20	50 ± 9	>200	>200	>200
NMR	300	ND^c^	200	200	100	200
ITC		300				

Measurements were not made for interactions where no K_D_ value is reported.

a For interactions measured using SPR, *Kᴅ* values are given as the geometric mean of a minimum of three independent measurements. For interactions measured using NMR, the *Kᴅ* values were determined from two or more peaks from a single titration and bootstrapped with 100 replicas in TITAN. For interactions measured using ITC, the *Kᴅ* values are given as the geometric mean of a minimum of two independent measurements. We estimate the uncertainty in each *K*_*D*_ to be ∼25% based on our experience measuring protein interactions. *Gray boxes* indicate affinities that were not measured.

b No binding (NB) observed.

c Not determined (ND) because of unreliable fits to the data.

Because of the limited solubility of the original peptides, we synthesized analogs that incorporated two glutamates at both the *N-* and *C-*termini of each peptide to improve solubility and facilitate further characterization of the peptide–BD interactions (Fig. 1*D*). The new peptides (1AcK.4E and 2AcK.4E) could be prepared at much higher concentrations than the original pair. The affinities of biotinylated variants of the glutamate-containing peptides for each BET BD were determined by immobilizing the peptides to a streptavidin-coated SPR chip and titrating with the BDs. The 2AcK.4E peptide bound BD1 domains with affinities of ∼20 to 70 μM and BD2 domains with affinities weaker than 200 μM (Fig. 2*B* and Table 1). As with the original peptides, the binding curves demonstrated fast association and dissociation kinetics. The glutamates thus improved solubility at the expense of affinity. Nevertheless, we could now measure binding of 1AcK.4E, which bound BD1 domains with affinities weaker than the corresponding interactions with 2AcK.4E. As none of the titrations against 1AcK.4E went to completion in our experiments, all affinities were estimated to be >200 μM (Fig. 2*C* and Table 1).

### Peptides bind in the canonical AcK-binding pocket

To investigate the binding mode of the peptides selected from our RaPID screen, we carried out ^15^N-heteronuclear single quantum coherence (HSQC) chemical shift perturbation (CSP) experiments using both 1AcK.4E and 2AcK.4E. Furthermore, to explore whether the addition of solubilizing glutamates had altered the peptide–BD binding mode, we also tested 2AcK, which was sufficiently soluble to perform these experiments using low concentrations of BDs (*e.g.*, by using ∼30 μM ^15^N-labeled BD, the addition of 8 molar equivalents of peptide could be achieved). ^15^N-HSQC spectra were recorded following each addition of peptide into uniformly ^15^N-labeled BRD3–BD1 or BRD3–BD2.

**Figure 3:**
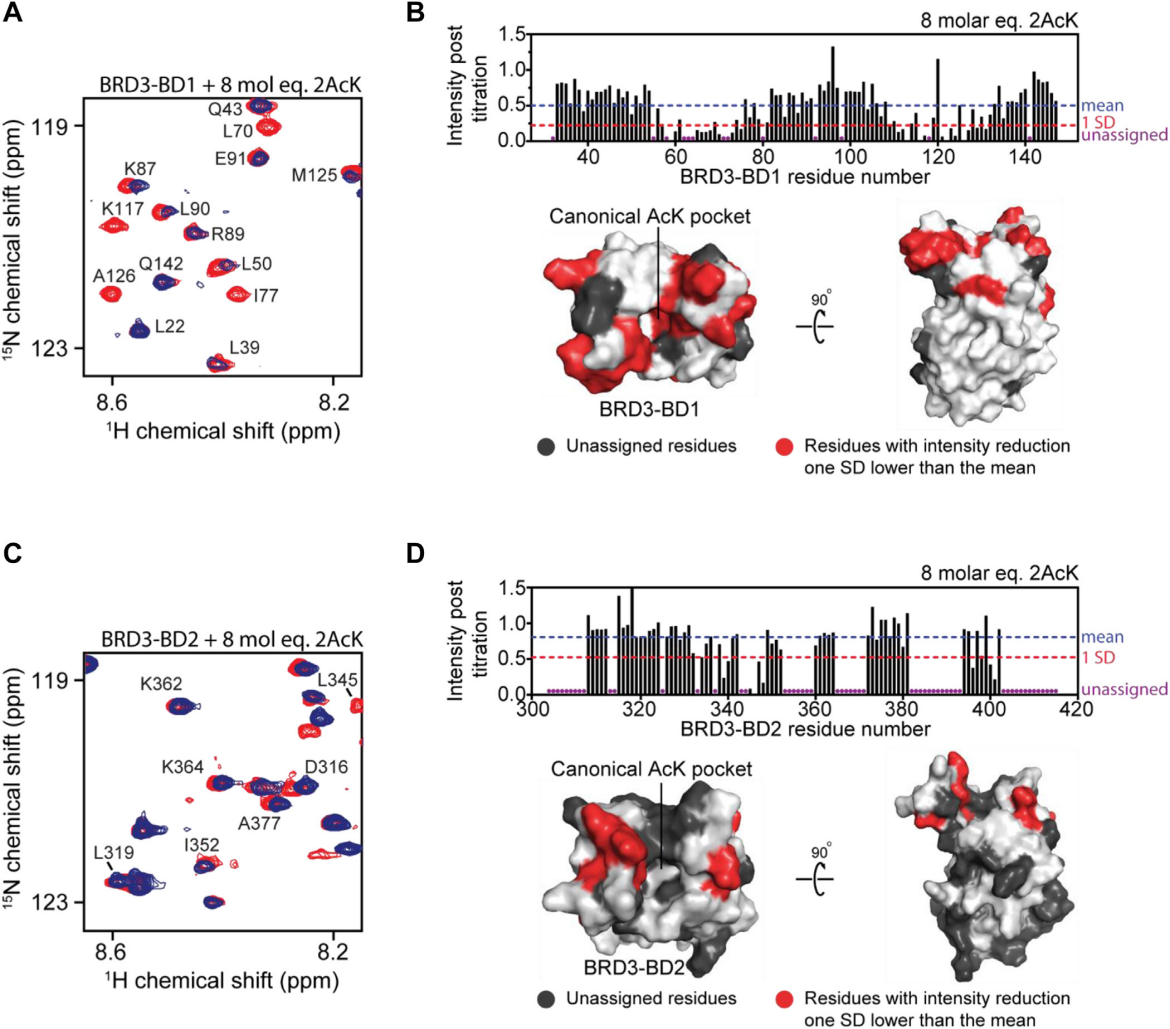
^**15**^**N-HSQC titrations for 2AcK into the BDs of BRD3 and structural mapping of the interactions.** For all ^15^N-HSQC titrations, BDs were used at a concentration of ∼30 μM, and the assignments of some of the signals are indicated. *A*, ^15^N-HSQC spectra of BRD3–BD1 alone (*red*) and in the presence of 8 molar equivalents of 2AcK (*blue*). *B*, *top*, quantitation of change in ^15^N-HSQC signal intensity for each signal following the addition of the indicated amount of 2AcK to BRD3–BD1. The mean intensity across all signals in the titration is marked by the *blue dashed line*, and the intensity reduction 1 SD below the mean is indicated by the *red dashed line*. Unassigned residues in the titration are indicated by *purple circles*. *Bottom*, residues for which signals are reduced in intensity by at least 1 SD from the mean reduction are mapped onto the structure of BRD3–BD1 (*light gray*) in *red*. Unassigned residues are colored *dark gray*. *C*, ^15^N-HSQC spectra of BRD3–BD2 alone (*red*) and in the presence of 8 molar equivalents of 2AcK (*blue*). *D*, *top*, quantitation of change in ^15^N-HSQC signal intensity for each signal following the addition of the indicated amount of 2AcK to BRD3–BD2. The mean intensity across all signals in the titration is marked by the *blue dashed line*, and the intensity reduction 1 SD below the mean is indicated by the *red dashed line*. Unassigned residues in the titration are indicated by *purple circles*. *Bottom*, residues for which signals are reduced in intensity by at least 1 SD from the mean reduction are mapped onto the structure of BRD3–BD2 (*light gray*) in *red*. Unassigned residues are colored *dark gray*. BD, bromodomain; HSQC, heteronuclear single quantum coherence.

In the titration of 2AcK into BRD3–BD1, widespread reductions in signal intensity—though with a range of magnitudes—were observed (Fig. 3*A*). The pattern of changes indicates that the interaction is in slow-to-intermediate exchange on the chemical shift timescale, consistent with the affinity observed by SPR. In contrast, and in line with the lower affinities measured by SPR, titrations with 2AcK.4E were in the intermediate-fast exchange regime, displaying perturbations to both signal intensity and position (Fig. S3*A*). To map the binding site of both peptides, we calculated the intensity of each signal after peptide addition as a fraction of the pre-titration intensity and plotted this value against residue number (Figs. 3*B* and S3*B*). Most changes were common to both peptides and mapping of the most significant changes (those greater than one standard deviation from the mean intensity change for each titration) onto the structure of BRD3–BD1 indicated a common BD-binding surface—one that directly overlaps the canonical AcK-binding pocket. Thus, both peptides bind to the native AcK-binding site and, although the introduction of flanking glutamates in 2AcK.4E reduces the affinity of the peptide, it does not alter its binding site.

In the titrations of 2AcK and 2AcK.4E against BRD3–BD2, substantially smaller CSPs were observed, indicating both interactions are in fast exchange on the chemical shift timescale and suggestive of a substantially weaker binding affinity for BRD3–BD2 than BRD3–BD1 (Figs. 3*C* and S3*C*). We quantified these changes in peak intensity and mapped the most significant changes onto the BRD3–BD2 structure. As for BRD3–BD1, these changes mapped to the canonical AcK-binding pocket on the BD2 (Figs. 3*D* and S3*D*).

The ^15^N-HSQC titrations of 1AcK.4E against BRD3–BD1 and BRD3–BD2 revealed CSPs to largely the same peaks observed in the corresponding titrations with 2AcK and 2AcK.4E (Fig. S4, *A* and *C*). However, fewer peaks changed, and the observed changes were smaller, in line with the weak (>200 μM) affinities measured for these interactions by SPR. Mapping the most significant intensity changes onto structures of BRD3–BD1 and BRD3–BD2 once again revealed binding largely to the same site on the BDs as observed for the 2AcK and 2AcK.4E (Fig. S4, *B* and *D*).

**Figure 4:**
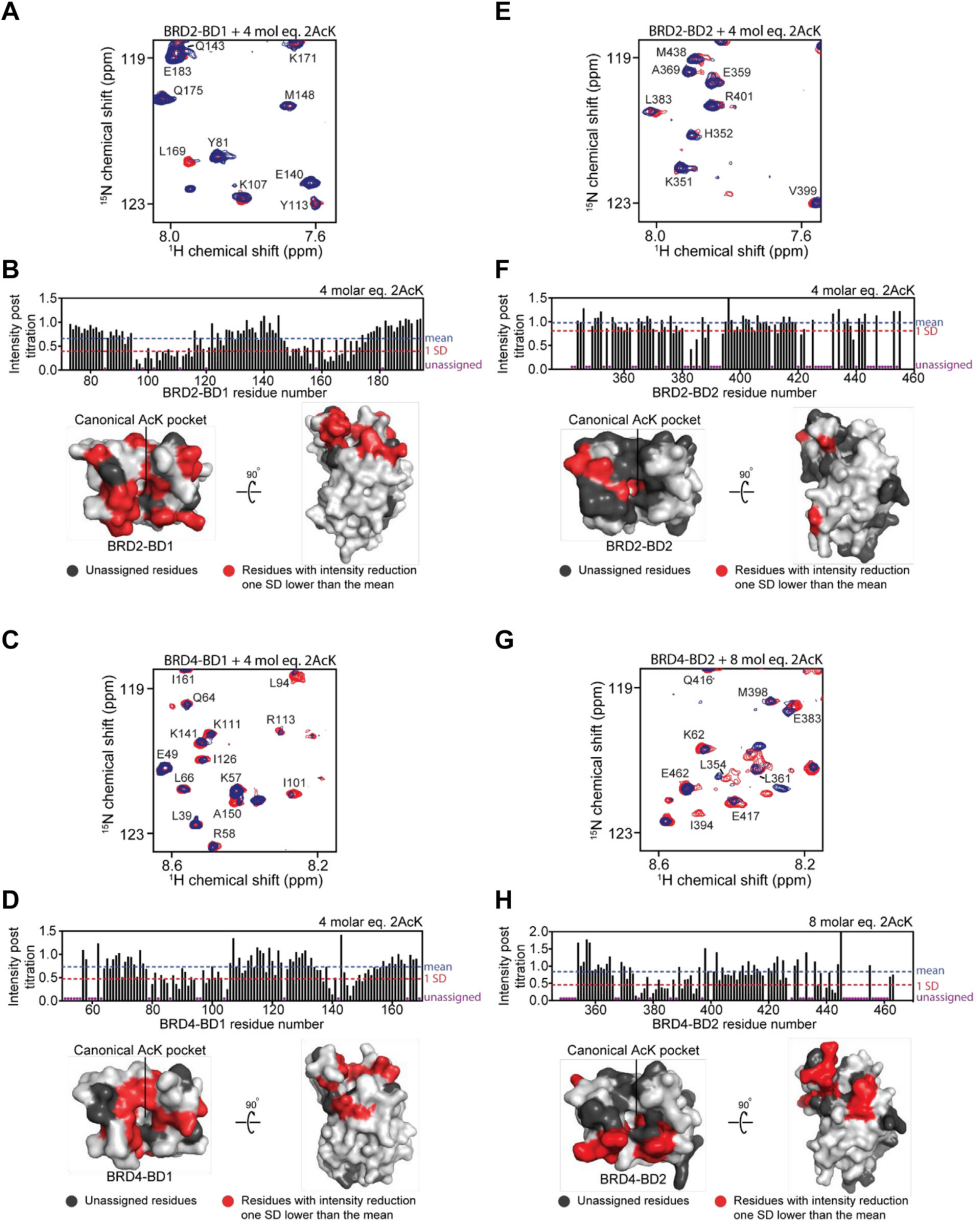
**Peptide 2AcK binds the BDs from BRD2 and BRD4 at the canonical binding site.** For all ^15^N-HSQC titrations, BDs were used at a concentration of ∼30 μM, and the assignments of some of the signals are indicated. *A*, ^15^N-HSQC spectra of BRD2–BD1 alone (*red*) and in the presence of 4 molar equivalents of 2AcK (*blue*). *B*, *top*, quantitation of change in ^15^N-HSQC signal intensity for each signal following the addition of the indicated amount of 2AcK to BRD2–BD1. The mean intensity across all signals in the titration is marked by the *blue dashed line*, and the intensity reduction 1 SD below the mean is indicated by the *red dashed line*. Unassigned residues in the titration are indicated by *purple circles*. *Bottom*, residues for which signals are reduced in intensity by at least 1 SD from the mean reduction are mapped onto the structure of BRD2–BD1 (*light gray*) in *red*. Unassigned residues are colored *dark gray*. *C*, ^15^N-HSQC spectra of BRD4–BD1 alone (*red*) and in the presence of 4 molar equivalents of 2AcK (*blue*). *D*, *top*, quantitation of change in ^15^N-HSQC signal intensity for each signal following the addition of the indicated amount of 2AcK to BRD4–BD1. The mean intensity across all signals in the titration is marked by the *blue dashed line*, and the intensity reduction 1 SD below the mean is indicated by the *red dashed line*. Unassigned residues in the titration are indicated by *purple circles*. *Bottom*, residues for which signals are reduced in intensity by at least 1 SD from the mean reduction are mapped onto the structure of BRD4–BD1 (*light gray*) in *red*. Unassigned residues are colored *dark gray*. *E*, ^15^N-HSQC spectra of BRD2–BD2 alone (*red*) and in the presence of 4 molar equivalents of 2AcK (*blue*). *F*, *top*, quantitation of change in ^15^N-HSQC signal intensity for each signal following the addition of the indicated amount of 2AcK to BRD2–BD2. The mean intensity across all signals in the titration is marked by the *blue dashed line*, and the intensity reduction 1 SD below the mean is indicated by the *red dashed line*. Unassigned residues in the titration are indicated by *purple circles*. *Bottom*, residues for which signals are reduced in intensity by at least 1 SD from the mean reduction are mapped onto the structure of BRD2–BD2 (*light gray*) in *red*. Unassigned residues are colored *dark gray*. *G*, ^15^N-HSQC spectra of BRD4–BD2 alone (*red*) and in the presence of 8 molar equivalents of 2AcK (*blue*). *H*, *top*, quantitation of change in ^15^N-HSQC signal intensity for each signal following the addition of the indicated amount of 2AcK to BRD4–BD2. The mean intensity across all signals in the titration is marked by the *blue dashed line*, and the intensity reduction 1 SD below the mean is indicated by the *red dashed line*. Unassigned residues in the titration are indicated by *purple circles*. *Bottom*, residues for which signals are reduced in intensity by at least 1 SD from the mean reduction are mapped onto the structure of BRD4–BD2 (*light gray*) in *red*. Unassigned residues are colored *dark gray*. BD, bromodomain; HSQC, heteronuclear single quantum coherence.

Because we observed more substantial peak perturbations in our titrations using 2AcK and 2AcK.4E compared with 1AcK.4E, we went on to explore the interactions of the doubly acetylated peptides with the BDs of BRD2 and BRD4. When compared with BRD3–BD1, similar sets of peaks in BRD2–BD1 and BRD4–BD1 were perturbed on addition of both peptides, though the overall magnitude of these changes was smaller (Figs. 4, *A*–*D* and S5, *A*–*D*). As observed with BRD3–BD1, the 2AcK peptide induced CSPs to the BD1s that were reminiscent of a slow-intermediate exchange regime, whereas 2AcK.4E displayed an intermediate-fast exchange regime with both signal intensity changes and movement observed. Of the peptide titrations against BD2 domains, the largest peak intensity and CSPs were observed with 2AcK titrated against BRD4–BD2 (Fig. 4, *G* and *H*), whereas titrations with 2AcK.4E resulted in comparable changes across all BD2 domains (Figs. S3, *C* and *D* and S5, *E* and *F*).

To corroborate the SPR-derived affinities, we sought to use the 2D lineshape analysis software TITAN to fit data from our ^15^N-HSQC titrations (18). Fits for titrations of 2AcK.4E into most of the BDs converged well, yielding *K*_*D*_ values that are broadly in line with the SPR-derived affinities (Table 1 and Fig. S6). However, titrations of BRD3–BD1 with either 1AcK.4E or 2AcK.4E did not converge, even when more complex models devised based on our structural data (see later) were used. This is likely because of the complexity of the linked equilibria that contribute to this interaction (the formation of an asymmetric 2:1 protein–peptide complex), which can often give rise to poorly defined minima in least-squares fitting calculations.

Taken together, our NMR titration data indicate that all three peptides bind all tested BDs in the canonical AcK-binding pocket, but with a range of affinities, and that the addition of solubilizing glutamates does not substantially alter this binding site.

### 2AcK binds BRD3–BD1 *via* an unprecedented mechanism

**Figure 5:**
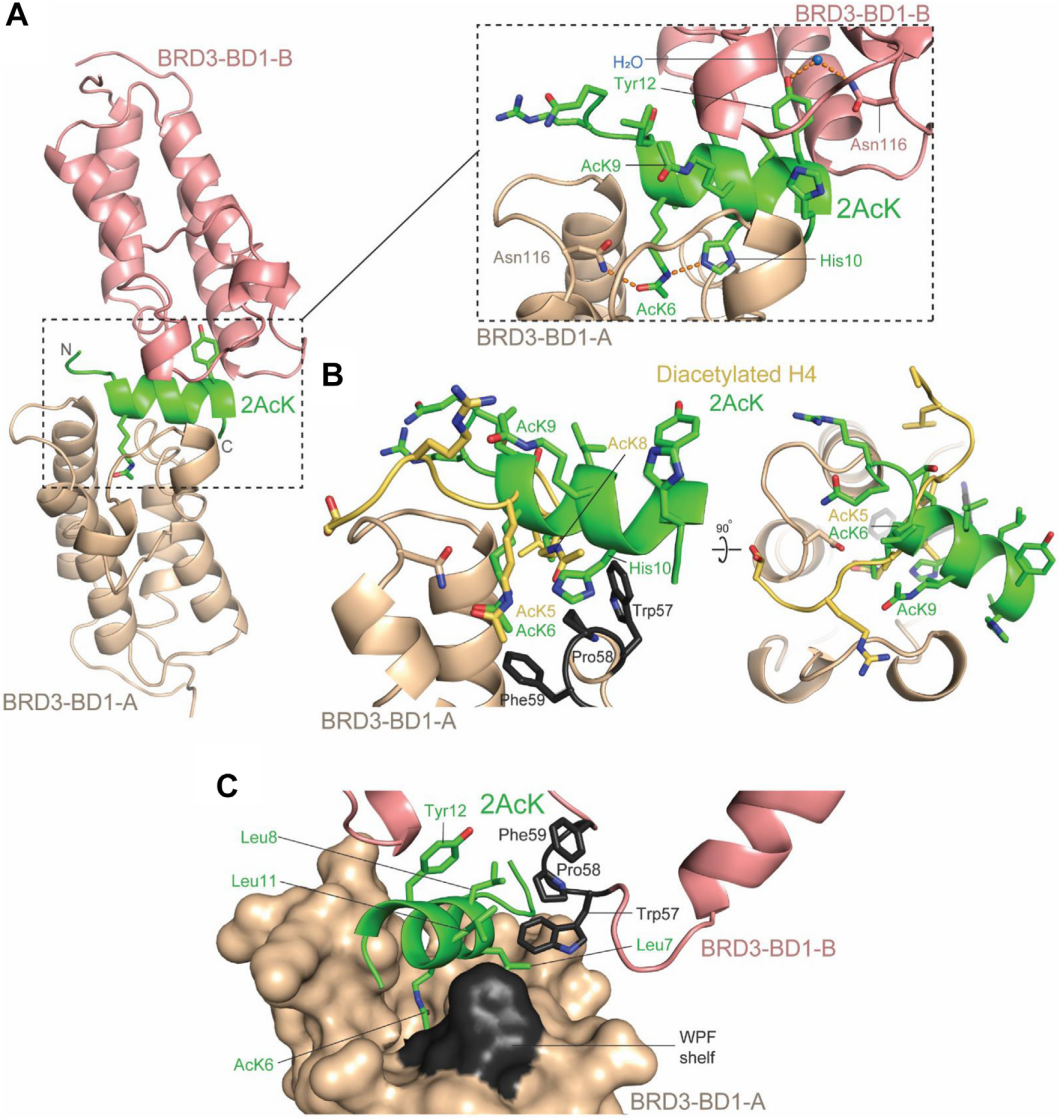
**Structural basis for the BRD3–BD1–2AcK interaction.***A*, structure of the BRD3–BD1–2AcK complex (PDB ID: 7TO8). The two molecules of BRD3–BD1 are shown in *wheat* (molecule A) and *salmon* (molecule B), and the peptide is shown in *green*. A close-up of the interaction is shown in the *dashed rectangular panel*. The two AcK residues are labeled, including AcK6 (which forms a hydrogen bond with Asn116 of BRD3–BD1-A), as is the Tyr12 residue that occupies the binding pocket of BRD3–BD1-B and forms a water-mediated hydrogen bond with Asn116 of that molecule. Hydrogen bonds are indicated by the *orange dashed lines*. *B*, comparison of the conformation taken up by 2AcK in the BRD3–BD1 complex with the conformation of a diacetylated histone H4 peptide (*yellow*) bound to BRD4–BD1 (PDB ID: 3UVW) (5). Views of the AcK insertion angle (*left*) and backbone conformation (*right*) are shown. *C*, interactions of leucine residues in 2AcK (*sticks*) with the WPF shelf (*black*) of both BRD3–BD1 molecules in complex with the peptide. BD, bromodomain; PDB, Protein Data Bank.

We next determined X-ray crystal structures for complexes formed between BRD3–BD1 and both 2AcK (1.5 Å resolution, Protein Data Bank [PDB] ID: 7TO8, Table S2) and 2AcK.4E (1.6 Å resolution, PDB ID: 7TO9, Table S2). In line with our NMR data, the structures of the BDs and peptides are almost identical with the exception that additional *N*- and *C*-terminal residues are visible for the 2AcK peptide (Fig. S7). Because of the similarity between the two structures, here we will describe only the BRD3–BD1–2AcK structure. As shown in Figure 5, two copies of BRD3–BD1 bind a single peptide. The two BDs take up an essentially identical conformation to each other and to other reported structures of the domain. The peptide forms two regular turns of α-helix, a conformation that has only been observed in BET-binding peptides for a diacetylated severe acute respiratory syndrome coronavirus 2 E-protein peptide in complex with BRD4–BD1 (PDB ID: 7TV0) but for no natural BD ligand (from a total of 39 structures; tabulated in Table S3). Interestingly, the two AcK residues of the severe acute respiratory syndrome coronavirus 2 E peptide, present at the opposing termini of the peptide, also each bind a separate molecule of BRD4–BD1 (19). The two BDs in the BRD3–BD1–2AcK structure are related by a twofold noncrystallographic axis that runs perpendicular to the long axis of the peptide α-helix (Fig. S8*A*). This rotation maps the helix directly onto itself, except in the opposite direction. Both orientations of the peptide were found equally in the crystal structure. The symmetry orientation also caused the two pocket-binding residues to swap their positions.

In the structure, one BD binds the *N*-terminal AcK (AcK6) of the peptide in the canonical pocket, whereas the corresponding pocket in the second BD is occupied by Tyr12 (Fig. 5*A*, *inset*, numbering is as in Fig. 1*D*). The acetyl group of AcK6 forms the same hydrogen bond with the side chain of Asn116 that is common to all characterized BD–peptide interactions. Likewise, the overall position of AcK6 in the pocket is the same as that observed for complexes with native ligands such as AcK5 of histone H4 (Fig. 5*B*). In the second BD, the hydroxyl group of Tyr12 forms a water-mediated hydrogen bond also to Asn116 (Fig. 5*A*, *inset*). The total buried surface area at the BD–peptide interface involving AcK6 is 1320 Å^2^ and 1010 Å^2^ for the interaction involving Tyr12, which is significantly larger than area buried in, for example, the complex formed between BRD4–BD1 and the diacetylated (on K5 and K8) histone H4 ligand (690 Å^2^; PBD ID: 3UVW; Fig. S9*B*) (5). In contrast to large surface contact between the peptide and each BD, the two BD chains make very few interdomain contacts, burying a total of only 260 Å^2^ of surface area.

In structures of BET-family BDs bound to diacetylated peptides from the native target histone H4 as well as the transcription factors GATA1, E2F1, and Twist (5, 7, 12), the peptide backbone is extended and runs at roughly 90° to the long axis of the 2AcK helix (Fig. 5*B*). This difference allows 2AcK to place a histidine (His10) into a portion of the binding pocket that is not utilized by native partners and furthermore to form a hydrogen bond with the Nε of the amide group of AcK6 (Fig. 5*A*, *inset*). An arrangement of this type is not possible with native ligands because of the position of the ligand backbone (Fig. 5*B*).

In complexes with diacetylated native ligands, the more *C*-terminal of the two AcK residues forms van der Waals interactions with the so-called “WPF” shelf on the edge of the binding pocket (Figs. 5*B* and S1*B*). In the 2AcK complex, however, the corresponding AcK (AcK9) occupies a very different position (because of the peptide helical conformation) and is not in contact with the WPF shelf of either of the two BDs (Fig. 5*B*). Instead, Leu7 and Leu11 form contact with the WPF shelf of the AcK6-binding BD (Figs. 5*C* and S8*B*). These residues, together with Leu8, simultaneously interact with the WPF shelf of the second BD. It is worth mentioning that positions of Leu7 and Leu11 are swapped because of the rotational symmetry of the complex. Finally, we note that side chains of residues *N* terminal to AcK6 were either not in contact with the BD or did not display any electron density, suggesting that they were likely not well ordered. This observation is consistent with the lack of sequence preference for these *N*-terminal residues.

Given the two distinct binding modes observed for 2AcK in the crystal structure, each present at equal occupancy, we conducted isothermal titration calorimetry (ITC) to assess if the peptide can simultaneously engage two BDs in solution. Titration of BRD3–BD1 into 2AcK revealed uniphasic exothermic binding curves, and fitting of the data to a binding isotherm yielded an N value of ∼2.5, consistent with the binding of two proteins, each with approximately equal affinity (Fig. S9*A*). A *K*_*D*_ of 10 ± 2 μM was calculated, ∼5 times weaker than the affinity measured for the interaction using SPR. As the BD was immobilized to a surface in the SPR experiment, the affinity measured using this technique might be enhanced because of avidity effects.

ITC was also used to quantify the interaction between BRD3–BD1 and 2AcK.4E (Fig. S9*B*). Significantly weaker binding was observed, and the data could only be fit by fixing the N value to 2.5 (informed by the BRD3–BD1–2AcK data). An estimated *K*_*D*_ of approximately 400 ± 2 μM was derived, ∼15-fold weaker than the *K*_*D*_ measured for 2AcK—again perhaps because of avidity effects in the SPR measurements.

This 2:1 binding mode could also explain the widespread reductions in intensity observed in our HSQC titrations; the significant change in rotational correlation time and consequent enhancement in transverse relaxation associated with the formation of this elongated complex would be expected to reduce signal intensities across the protein.

### The 1AcK.4E binding mode partially mimics 2AcK and previously discovered unnatural BD–peptide ligands

**Figure 6:**
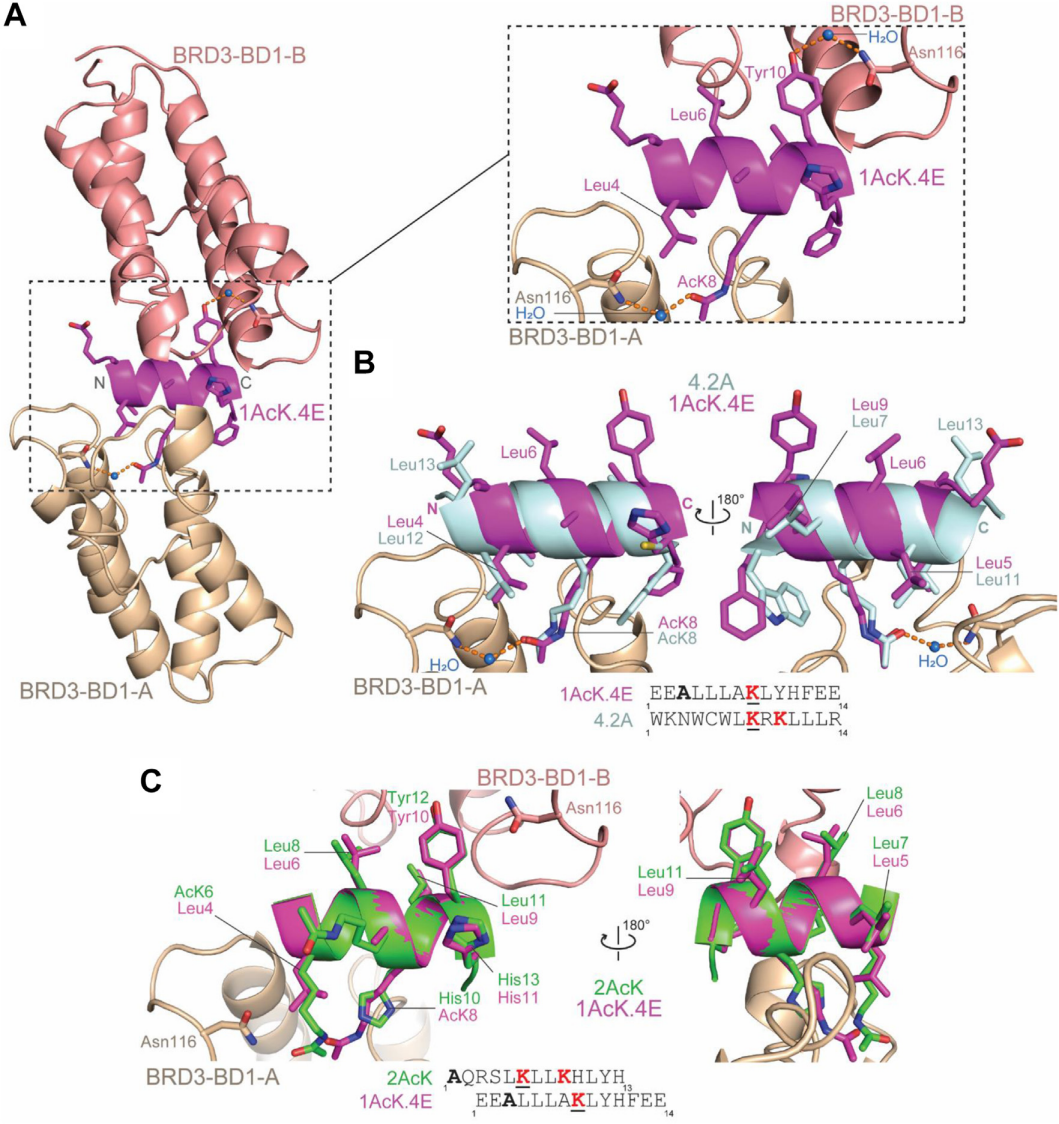
**Structural basis for the BRD3–BD1–1AcK.4E interaction.***A*, structure of the BRD3–BD1–1AcK.4E complex (PDB ID: 7TO7). The two copies of BRD3–BD1 are shown in *wheat* (*A*) and *salmon* (*B*), and the peptide is shown in *magenta*. A close-up of the interaction is shown in the *dashed rectangular panel*. AcK8 and Tyr10, which each occupy the binding pocket of one of the BDs, are labeled, as is the water molecule (*blue sphere*) that forms hydrogen bonds bridging Asn116 and the pocket binding residue (either AcK8 or Tyr10). The hydrogen bonds are indicated by the *orange dashed lines*. *B*, structure of the peptide 4.2 A (*pale cyan*; crystallized in complex with BRD4–BD1, PDB ID: 6ULV) overlayed onto the structure of BRD3–BD1-A–1AcK.4E (14). The angle of entry of AcK8 in 4.2 A into the binding pocket mirrors that of AcK8 in 1AcK.4E. The overlay shows the conserved locations of leucine residues that contact the WPF shelf (Fig. S8*B*). Leu7 and Leu11 of 4.2 A map onto Leu9 and Leu5 of 2AcK, respectively. *C*, overlay of 1AcK.4E (*magenta*) with 2AcK (*green*). The backbone conformations, positions, and identities of many side chains are closely matched despite the differing positions of the pocket-binding AcK residues. The *underlined red* AcK residues are those that engage the canonical binding pocket. BD, bomodomain; PDB, Protein Data Bank.

We also determined the X-ray crystal structure of BRD3–BD1 bound to 1AcK.4E (1.9 Å resolution, PDB ID: 7TO7, Table S2). The overall architecture of the complex closely resembles the BRD3–BD1–2AcK structure with two BDs binding a helical peptide and all three chains in the same relative spatial arrangement (Fig. 6*A*). The two copies of the BD are again related by a two-fold noncrystallography axis observed for the 2AcK structures. Again, the electron density for the peptide is best modeled at 50% occupancy in the two symmetrical orientations (Fig. S10*A*). Furthermore, the binding pocket of one BD accommodates the AcK8 residue, whereas the other binds Tyr10 (Figs. 1*D*, 6*A* *inset*, and S10*B*). Compared with the 2AcK structures, slightly less surface area is buried while forming the complex (1100 and 1000 Å^2^ for the AcK-mediated and Tyr-mediated interactions, respectively, and 260 Å^2^ between the two BDs).

Although the 1AcK.4E sequence differs significantly from 2AcK and sequence distance between the two pocket-binding residues (AcK8 and Tyr10) is smaller compared with 2AcK (AcK6 and Tyr12), the overall position of the peptide backbone remains the same. This change in relative position of the binding pocket residues, which are closer by one α-helix turn in 1AcK.4E, is accommodated by the different conformation of AcK8 in the binding pocket (Fig. 6*C*). We have observed this “diagonal” conformation in helical cyclic peptides (such as 4.2A) discovered previously in RaPID screens against BET BDs (11) (Fig. 6*B*). In parallel with those structures, AcK8 of 1AcK.4E forms a water-mediated hydrogen bond with Asn116 (as does Tyr10; Fig. 6*A*, *inset*).

Comparison of the 1AcK.4E and 2AcK structures reveals that five residues make essentially identical interactions in the two structures (Fig. 6*C*): the pocket-binding tyrosine (Tyr10 in 1AcK.4E; Tyr12 in 2AcK), the three leucines that pack against the WPF shelf (Leu5, Leu6, and Leu9 in 1AcK.4E; Leu7, Leu8, and Leu11 in 2AcK), and the histidine that immediately follows the tyrosine (His11 in 1AcK.4E; His13 in 2AcK). Strikingly, 4.2A also displays two of the three leucines in the same positions (Leu7 and Leu11), with the aliphatic portion of an AcK side chain occupying the third position to pack against the WPF shelf (Fig. 6*B*). It is also notable that this similarity in positioning of these residues occurs despite the fact that the 4.2A helix runs in the opposite direction to the peptides discovered in this study (Fig. 6*B*).

Finally, we assessed the conformational preferences of 2AcK.4E by NMR spectroscopy. Proton chemical shifts are most consistent with the peptide being intrinsically disordered, and two-dimensional NOESY and rotating frame Overhauser effect spectroscopy data display very few of the NOE patterns (*e.g.*, an extended series of HN–HN NOEs between sequential residues) that are diagnostic of helical structure (Fig. S11). It is therefore likely that the helical structure observed in the complexes arises from conformational selection from a rarely populated conformer.

## Discussion

Reported natural ligands of the BET BDs have diverse sequences—the full extent of which are still unknown—and dissociation constants ranging from tens to hundreds of micromolar. In contrast, we recently derived a set of cyclic peptide ligands that bind to BET BDs with binding affinities in the nanomolar and high picomolar ranges (11). In the current study, we therefore sought to explore (a) the range of sequence contexts that could support high-affinity binding of acetylated lysines to BET BDs and (b) whether linear sequences could also achieve higher affinities than known biological ligands and therefore might represent a new class of natural BET BD targets.

We therefore devised and tested a strategy that we hypothesized could be used to deliver a more comprehensive view of preferred BD-binding motifs. We used RaPID mRNA display to screen two peptide libraries, each comprising ∼10^12^ peptides and harboring either one or two AcK residues. The AcKs were separated by two residues to mimic the arrangement found most frequently in natural diacetylated BET ligands. Although we envisaged that our screens might provide a broad overview of the substrate preferences of BRD3–BD1, each screen converged rapidly on one major consensus sequence—and these sequences were similar though not identical between the two screens. Despite what appeared to be an unusually strong preference for these sequences, their affinities for BET BDs (as high as ∼1 μM for 2AcK) were still several orders of magnitude weaker than those observed for our previously identified cyclic peptides. The affinity of 2AcK for BRD3–BD1 was nonetheless higher than all other measured natural ligands.

Unexpectedly, however, a search for these sequences in the human proteome using BLASTp (National Center for Biotechnology Information), or even for the “core” sequences KLLKHLYH (from 2AcK) and LLLXKLYHF (from 1AcK, where X can be any amino acid), revealed no exact matches. Although this observation is not definitive evidence that the sequence has been selected against during evolution, it is nonetheless surprising given the large number of proteins to which the BET BDs have been shown to bind.

### A new BET BD–binding mode for linear peptides

The binding modes of our selected peptides also differed significantly from previously characterized natural ligand–BET BD binding interactions. A structure of 2AcK bound to BRD3–BD1 revealed that, although the more *N*-terminal AcK binds with a conformation closely resembling the corresponding residue in native substrates, the second AcK contacts a completely different surface (Fig. 5). The size of the interface made with each BD is also substantially greater than that observed for native partners with as many as nine residues from the peptide contacting both BDs in the crystal structure.

Both screens yielded peptides that formed a regular α-helix. This conformation is not adopted by any known endogenous BET target sequences, although several of our previously isolated cyclic peptide ligands have helical structures (11). In all cyclic peptides that we structurally characterized, the helix backbone overlays closely with the helix observed in this current study, though these cyclic peptide helices run in opposite directions in different peptides. Strikingly, bidirectionality of binding to BDs within a *single* peptide, as observed in this study, is new. Furthermore, the pocket-binding AcK in all previously discovered helical peptides had the “diagonal” entry angle observed for 1AcK.4E in the current study—in contrast to the perpendicular angle observed for the 2AcK peptide and for our tightest-binding cyclic peptides.

The affinity of 2AcK for BET-BD1 domains is lower than the affinities observed for our helical cyclic peptides by a factor of ∼50 (11), broadly suggesting that macrocycle formation can boost the achievable affinity for this interaction modality by one to two orders of magnitude, most likely because of the reduction in conformation entropy that needs to be lost upon complex formation. This idea is consistent with our observation that 2AcK.4E is largely disordered in solution.

### Screening with a monoacetylated library suggests that positioning of the AcK in the helix is important for high-affinity binding

The library design for our 1AcK screen placed the “warhead” AcK near the center of the peptide: residue 6 in a 10-residue sequence. This arrangement precluded the selection of peptides with the same architecture as 2AcK, because all seven residues *C*-terminal to the pocket-binding AcK in 2AcK make direct contact with the BDs, and only four residues are available in the 1AcK peptide. Instead, the most highly selected sequences from the 1AcK screen retain five of the key binding residues (Leu7, Leu8, Leu11, Tyr12, and His13) in the same relative positions but “shift” the AcK in the *C*-terminal direction by one helical turn.

The resulting diagonal entry angle for this AcK leads to formation of a water-bridged hydrogen bond between the AcK and Asn116, in contrast to the direct hydrogen bond formed by 2AcK. Comparison of the affinities measured for 1AcK.4E and 2AcK.4E suggests that the difference in AcK binding mode is worth at least threefold in affinity for binding to BD1 domains. In contrast, binding to BD2 domains is similar for 1AcK and 2AcK peptides, although the structural basis for this distinction is not clear.

Unexpectedly, both the 1AcK and 2AcK peptides also use a Tyr to partially occupy the binding pocket of the second BD in the complex. While there are previous instances of Tyr in the binding pocket, all examples had a “primary” AcK in the pocket as well with the Tyr stabilizing this AcK *via* a water-mediated bridge (10). The direct mode of Tyr interaction with Asn116 we report here, albeit through a water-mediated hydrogen bond, has not been described before. Again, it is noteworthy that this mode of interaction with Asn116 is mirrored by the AcK in the 1AcK peptide here and also the AcK in the 4.2A cyclic peptide from (11)—raising the possibility that such interactions might occur *in vivo*, though most likely with lower affinity.

In conclusion, given that the affinity observed for 2AcK is markedly stronger than that observed for many known natural ligands of BET proteins, our data suggest that lower affinities might be better suited to the signaling role played by these proteins. Similar arguments have been made for other protein-based signaling systems, and high-throughput screening approaches such as mRNA display represent a useful tool for exploring such hypotheses.

## Experimental procedures

### Protein expression and purification

The BDs from human BRD2 (BD1: 65–194; BD2: 347–455), BRD3 (BD1: 25–147; BD2: 307–419), and BRD4 (BD1: 42–168; BD2: 348–464) were expressed in *Escherichia coli* from pQE80L-Navi (Qiagen) as *N*-terminally His-tagged and biotinylated proteins for SPR. For other experiments, they were expressed from pGEX-6P (Cytiva) as *N*-terminal glutathione-*S*-transferase-fusion proteins. Expression and purification, *via* affinity chromatography and size-exclusion chromatography, of unlabeled and uniformly ^15^N-labeled proteins were carried out as described previously (11).

### RaPID screening

We synthesized two linear libraries from oligos purchased from Eurofins Genomics K.K. Each DNA sequence contained the standard RaPID T7 polymerase–binding site, a ribosome-binding site, a randomized peptide-coding region, a (Gly-Ser)_3_ linker, an amber stop codon, and a sequence for puromycin ligation. These libraries were prepared following standard protocols, transcribed to RNA, and ligated with puromycin for use in the two selections.

1AcK library: TAATACGACTCACTATAGGGTTGAACTTTAAGTAGGAGATATATCCATG(NNW)_4_ATG(NNW)_4_GGGTCTGGGTCTGGGTCTTAGGTAGGTAGGCGGAAA

2AcK library: TAATACGACTCACTATAGGGTTGAACTTTAAGTAGGAGATATATCCATG(NNW)_4_ATG(NNW)_2_ATG(NNW)_4_GGGTCTGGGTCTGGGTCTTAGGTAGGTAGGCGGAAA

Using these linear libraries, RaPID screens were performed as previously reported (11). The first-round translation was performed on an 80 μl scale and subsequent rounds on a 5 μl scale. For codon reprogramming, the 3,5-dinitrobenzyl ester of *N*^ε^-acetyl-lysine was synthesized as previously described and aminoacylated onto tRNA^Asn^_CAU_ using dFx (2 h, room temperature [RT], standard aminoacylation conditions) whilst tRNA^fMet^_CAU_ was aminoacylated with *N*-(acetoxy)-l-alanine (Ac-L-Ala), *via* the 3,5-dinitrobenzyl ester using dFx (72 h, 4 °C, standard aminoacylation conditions). Flexizymes were prepared as described previously (20).

Peptide sequences from deconvoluted Illumina sequencing data are presented in Table S1.

### Peptide synthesis

Peptides 2AcK and 1AcK were purchased from Ontores. Peptides 2AcK.4E and 1AcK.4E were synthesized as *C*-terminal amides using standard fluorenylmethyloxycarbonyl (Fmoc)-strategy solid-phase chemistry using an SYRO I automated synthesizer (Biotage). Fmoc-protected amino acids (Fmoc-Xaa-OH), coupling reagents, and resins were purchased from Mimotopes or Novabiochem. *N,N*-dimethylformamide (DMF) for peptide synthesis was purchased from RCI.

The resin (89 mg, 50 μmol, 0.56 mmol g^−1^, 1 eq.) was treated with 40% piperidine in DMF (800 μl) for 4 min, drained, then treated with 20 vol.% piperidine in DMF (800 μl) for 4 min, drained, and washed with DMF (4 × 1.6 ml). The resin was then treated with a solution of Fmoc-Xaa-OH or chloroacetic acid (200 μmol, 4 eq.) and Oxyma (220 μmol, 4.4 eq.) in DMF (400 μl), a 1 wt.% solution of 1,3-diisopropyl-2-thiourea in DMF (400 μl), followed by a solution of DIC (200 μmol, 4 eq.) in DMF (400 μl). Coupling of Fmoc-Cys(Trt)-OH and Fmoc-His(Trt)-OH was carried out at 50 ºC for 30 min. All other coupling reactions were conducted at 75 ºC for 15 min. The resin was then drained and washed with DMF (4 × 800 μl) before being treated with a solution of 5 vol.% Ac_2_O and 10 vol.% *i*Pr_2_NEt in DMF (800 μl) for 6 min at RT, drained, and washed with DMF (4 × 800 μl).

The resin was thoroughly washed with CH_2_Cl_2_ (4 × 7 ml) before being treated with 90:5:5 v/v/v TFA:triisopropylsilane:H_2_O and shaken at RT for 2 h. The resin was filtered, and the filtrate concentrated under a stream of nitrogen before addition of diethyl ether (40 ml). The peptide was pelleted by centrifugation (3 min, 2 ºC, at 6500 RCF), and the diethyl ether was decanted.

Preparative reversed-phase HPLC was performed using a Waters 600E multisolvent delivery system with a Rheodyne 7725i injection valve (5 ml loading loop) with a Waters 500 pump and a Waters 490E programmable wavelength detector operating at 214 and 280 nm. Preparative reversed-phase HPLC was performed using a Waters X-Bridge C18 OBD Prep Column (5 μm, 19 × 150 mm) at a flow rate of 15 ml min^−1^ using a mobile phase of 0.1% TFA in water (solvent A) and 0.1% TFA in MeCN (solvent B) on linear gradients, unless otherwise specified. Purity of peptides was determined by mass spectrometry recorded on a Shimadzu 2020 (electrospray ionization) mass spectrometer operating in positive and negative modes (Figs. S12 and S13). Gradient grade acetonitrile (MeCN) for liquid chromatography was purchased from Merck.

The concentrations of the peptides used in this study were determined by measuring absorbance at 205 nm (21) and NMR (22) quantification using a known concentration of 4,4-dimethyl-4-silapentane-1-sulfonic acid as a reference. Peptides were first dissolved in either water or experimental buffer, and measurements were made on the soluble peptide remaining in solution following removal insoluble peptide *via* centrifugation.

### SPR

Measurements were conducted on a T200 (Cytiva) and data analyzed using the Biacore Insight Evaluation Software. Experiments were performed at 4 °C in multicycle kinetics mode and fit using the equilibrium steady state affinity model (1:1 Langmuir binding isotherm). For experiments measuring the binding of the 2AcK peptide, biotinylated BET BDs were immobilized on a biotin CAP chip (Cytiva) with a target density of ∼1000 to 2000 RU and peptide was flowed over the chip. A buffer comprising 20 mM Mes (pH 6.5), 150 mM NaCl, 0.05% (v/v) Tween-20 was used as the running buffer. For experiments measuring the binding of 1AcK.4E and 2AcK.4E, *C*-terminally biotinylated variants of the peptides were immobilized on a CM5 chip (Cytiva), which had been amine-coupled with streptavidin, to a target density of ∼50 to 100 RU and unlabeled BET BDs were flowed over the chip. A buffer comprising 20 mM Hepes (pH 7.5), 150 mM NaCl, and 0.05% (v/v) Tween-20 was used as the running buffer.

### NMR spectroscopy

NMR samples of BET BDs were prepared at ∼25 to 100 μM for peptide titrations. Spectra were acquired at 298 K using Bruker Avance III 600 or 800 MHz NMR spectrometers fitted with TCI probe heads and using standard pulse sequences from the Bruker library. TOPSPIN3 (Bruker) and NMRFAM-SPARKY were used for spectral analysis (23). Spectra were internally referenced to 10 μM 4,4-dimethyl-4-silapentane-1-sulfonic acid. CSP experiments were performed by collecting ^15^N-HSQC spectra of ^15^N-labeled BDs before and after titration of unlabeled peptide into the samples.

Chemical shift assignments were taken from previous work (11) and CSP plots were generated using the following approach. A reference HSQC was recorded without the ligand present (ref HSQC), and then the ligand was added and a second HSQC (shift HSQC) recorded. The two HSQCs were peak picked using the APES algorithm in NMRFAM-Sparky to generate peak lists. The distance between each peak on the ref HSQC and all peaks on the shift HSQC was computed as follows:

$$dist=\sqrt{{\left({\delta H}_{ref}-{\delta H}_{shift}\right)}^{2}+{0.1*\left(\delta N_{ref}-{\delta N}_{shift}\right)}^{2}}$$

For intensity-based plots, peak height values were extracted from the ref HSQC and shift HSQC, and the values were normalized against values from peaks that were invariant throughout the titration. The intensities post titration were plotted against residue number, and the mean intensity across each titration was calculated. Residues with intensities 1 standard deviation lower than the mean intensity were considered significant interactions and plotted onto the structures of the BET BDs.

### X-ray crystallography

All crystallization studies described in this study were performed at 18 ºC using commercial 96-well crystallization screens in a sitting-drop vapor-diffusion format. Purified BRD3–BD1 (15 mg/ml) was mixed with ∼5 molar equivalents of peptide, and the complexes were incubated for 0.5 h before being dispensed into MRC two-drop chamber, 96-well crystallization plates using a Mosquito crystallization robot (TTP Labtech). Complex:precipitant ratios of 1:1 and 2:1 were trialled ensuring that a final drop volume of 300 nl was maintained. Crystals of the BRD3–BD1–peptide complexes typically appeared within 1 to 2 days after setting up the experiments. The BRD3–BD1–2AcK complex crystallized in a solution containing 0.1 M MMT (pH 6.0) and 25% (w/v) PEG1500. The BRD3–BD1–2AcK.4E complex crystallized in a solution containing 0.1 M Hepes (pH 7.5), 0.2 M NaCl, and 10% (v/v) 2-propanol. The BRD3–BD1–1AcK complex crystallized in the solution containing 0.1 M PTCP (pH 4.0) and 25% (w/v) PEG1500. Single crystals were cryoprotected using 10% (v/v) glycerol mixed in with the solution that yielded the crystals and plunge-frozen in liquid nitrogen.

X-ray diffraction data were collected at the Australian Synchrotron using the Macromolecular Crystallography MX2 beamline (microfocus) at 100 K using a wavelength of 0.9537 Å (24). The autoprocessed data from the Australian Synchrotron (processed using the XDS pipeline) were further processed using AIMLESS from the CCP4i suite (CCP4 suite) (25, 26). Initial phases were obtained using PhaserMR to perform molecular replacement with the structure of BRD3–BD1 from PDB ID: 3S91 as a molecular replacement model (27). COOT (MRC-LMB) software was used for manual building of the peptides (28). For all structures described in this, initial attempts to build the peptides revealed additional density that corresponded to an additional copy of the peptides rotated ∼180° around the axis perpendicular to the α-helical backbone of the peptides. The best phases were obtained by modeling the two rotated copies of the peptides into the density at 50% occupancy each. The structures were refined using iterative rounds of manual model building in COOT followed by refinement using Phenix.refine (29). The data collection and refinement statistics for all structures described in this study are outlined in Table S2. The final models were deposited to and validated by the PDB. All structure diagrams presented in this article are generated using PyMOL (Schrödinger). Buried surface areas were determined using the PISA server (https://www.ebi.ac.uk/pdbe/pisa/; (30)).

### ITC

The affinities and stoichiometry for the interaction of BRD3–BD1 with 2AcK and 2AcK.4E peptides were determined using a MicroCal PEAQ ITC (Malvern Instruments). Titrations of BRD3–BD1 (1 mM) into peptide (25–50 μM) were carried out at 25 °C using a 0.2 μl initial injection and 20 to 24 subsequence 1 to 3 μl injections. Data were analyzed using Malvern MicroCal PEAQ ITC Software, using a protein into buffer baseline correction and fitted with a one-site binding model.

## Data availability

The coordinates and structure factors for all structures described in this article have been deposited in the PDB. The PDB IDs for all structures described in this study are as follows: 7TO8 (BRD3–BD1 in complex with 2AcK), 7TO9 (BRD3–BD1 in complex with 2AcK.4E), 7TO7 (BRD3–BD1 in complex with 1AcK.4E). The PDB ID codes for each structure have also been provided throughout the main text and are also listed in in the supporting information section.

## Supporting information

This article contains supporting information provided as a separate file.

## Conflict of interest

The authors declare that they have no conflicts of interest with the contents of this article.
