# Supplementary material for: mRNA display reveals a class of high-affinity bromodomain-binding motifs that are not found in the human proteome
Source: J Biol Chem. 2023 Nov 20;299(12):105482. doi: 10.1016/j.jbc.2023.105482 (PMC10758951; doi:10.1016/j.jbc.2023.105482)
Supplement: Supporting data [file mmc1.docx]

**SUPPORTING INFORMATION**

mRNA display reveals a class of high-affinity bromodomain-binding motifs that are not found in the human proteome

Jason K K Low^1, #^, Karishma Patel^1, #^, Natasha Jones^1^, Paul Solomon^1^, Alexander Norman^2^, Joshua W C Maxwell^2^, Petr Pachl^1^, Jacqueline M Matthews^1^, Richard J Payne^2^, Toby Passioura^1,3^, Hiroaki Suga^3^, Louise J Walport^3,4,5,*^, Joel P Mackay^1,*^

^1^School of Life and Environmental Sciences, University of Sydney, NSW 2006 Australia

^2^School of Chemistry, University of Sydney, NSW 2006 Australia

^3^Department of Chemistry, Graduate School of Science, The University of Tokyo, Hongo, Bunkyo-ku, Tokyo, 113-0033, Japan

^4^The Francis Crick Institute, 1 Midland Road, London NW1 1AT

^5^Department of Chemistry, Molecular Sciences Research Hub, Imperial College London, London W12 0BZ, UK

^#^These authors contributed equally to this work.

*To whom correspondence should be addressed:

JPM: joel.mackay@sydney.edu.au

LJW: louise.walport@crick.ac.uk

**LIST OF MATERIALS INCLUDED**

**INCLUDED IN THIS DOCUMENT:**

- **Supplementary Tables S2 – S3**
- **Supplementary Figure S1 – S13**

**PROVIDED AS SEPARATE FILES:**

**Table S1. Enriched sequences from four rounds of RaPID screening of the 1AcK and 2AcK libraries against BRD3-BD1.**

**Table S2.** Data collection and refinement statistics for the crystal structure of BRD3-BD1 in complex with 2AcK (PDB ID: 7TO8), BRD3-BD1 in complex with 2AcK.4E (PDB ID: 7TO9), and BRD3-BD1 in complex with 1AcK.4E (PDB ID: 7TO7) solved using molecular replacement.


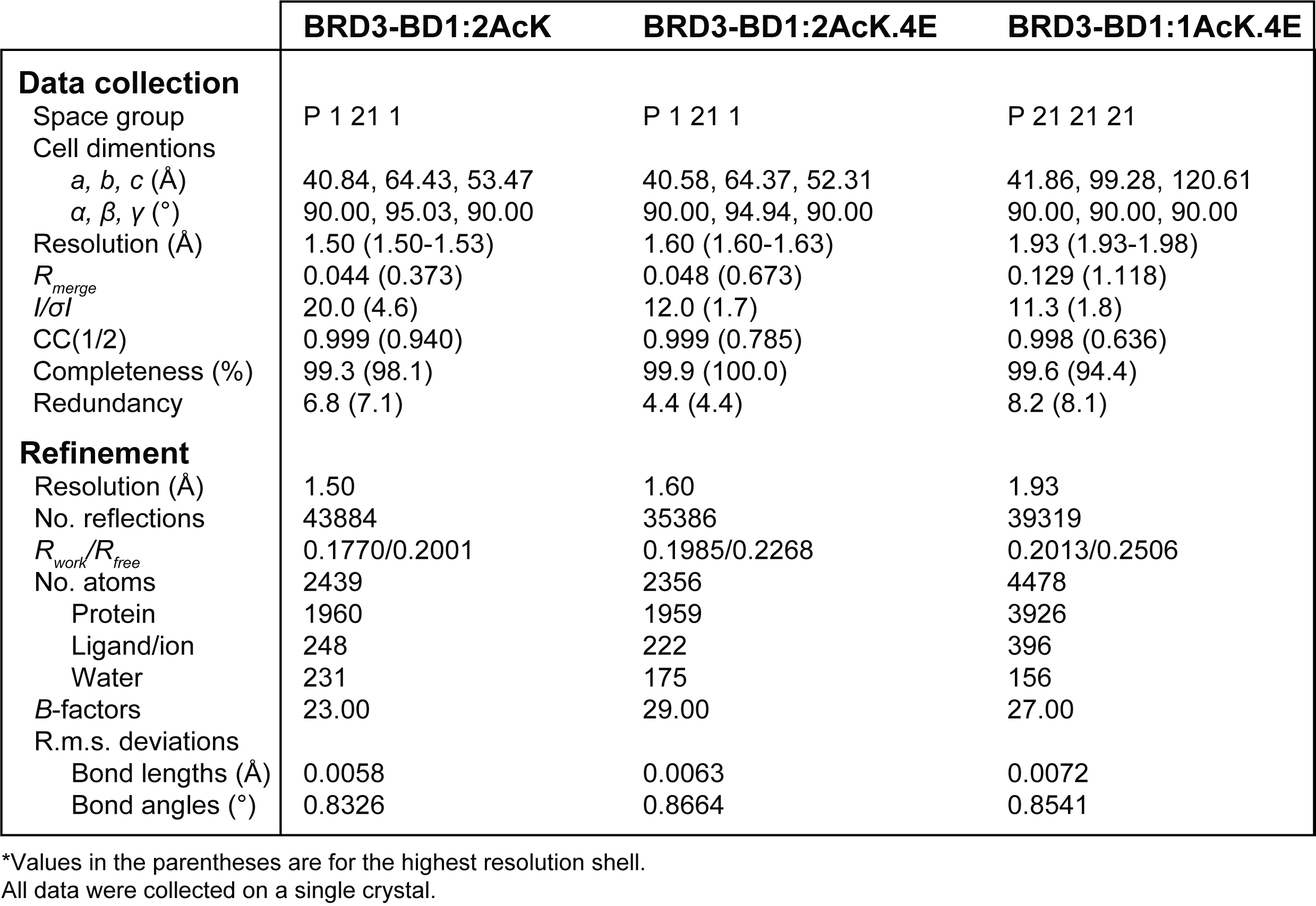


**Table S3.** Structures of all BET BD (grey):acetylated natural ligand (red) structures available in the PDB.

**
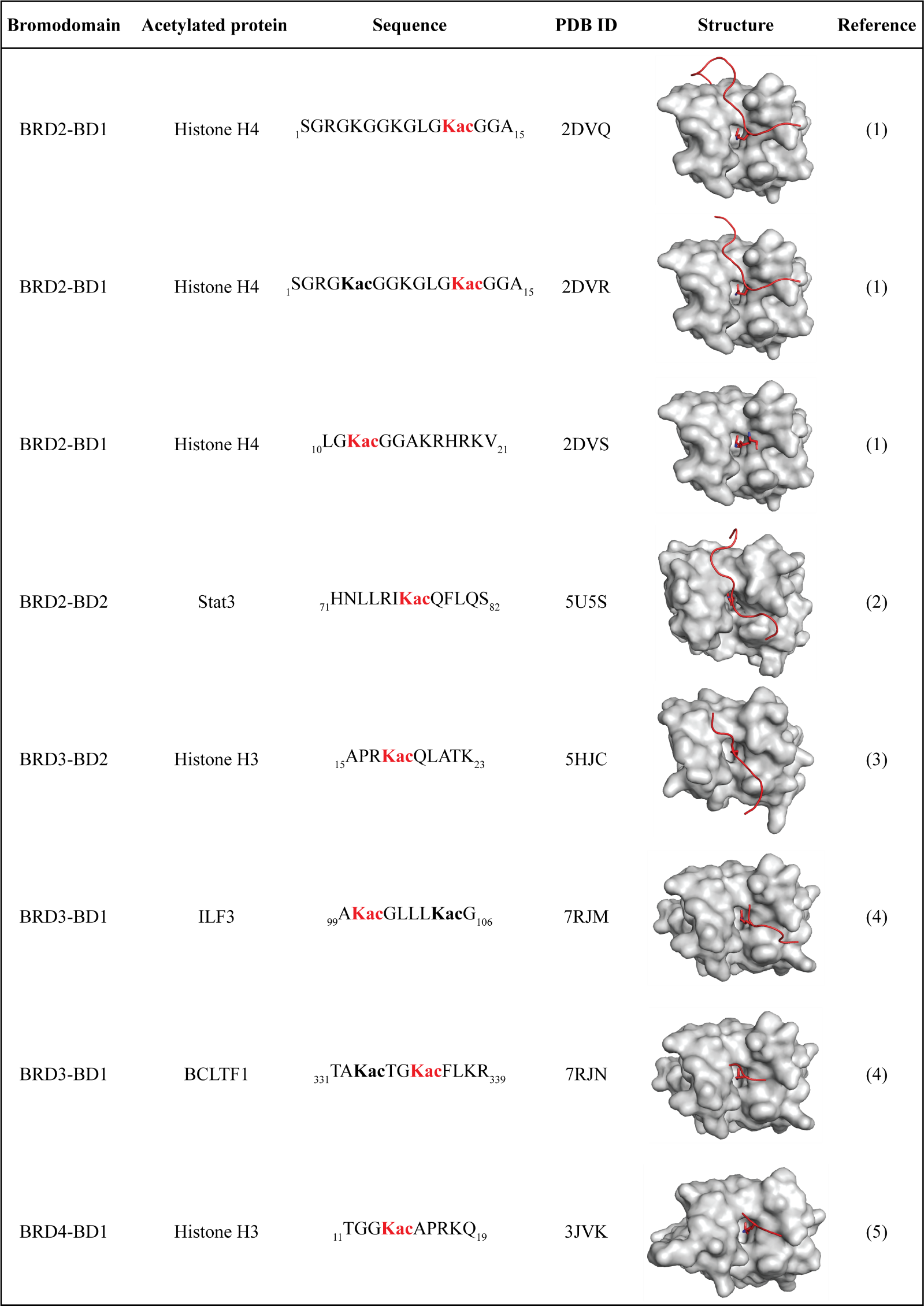
**

**
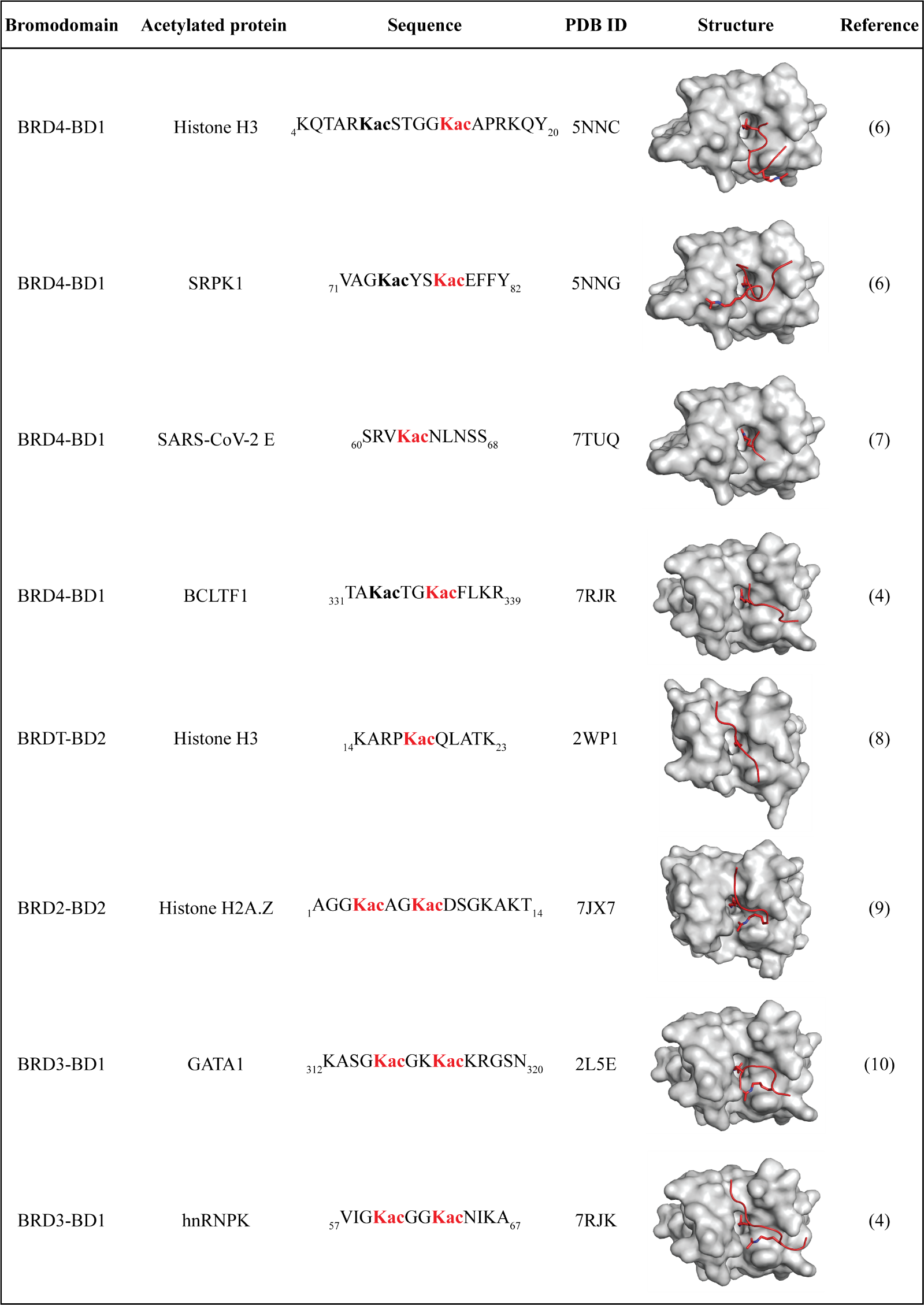
**

**
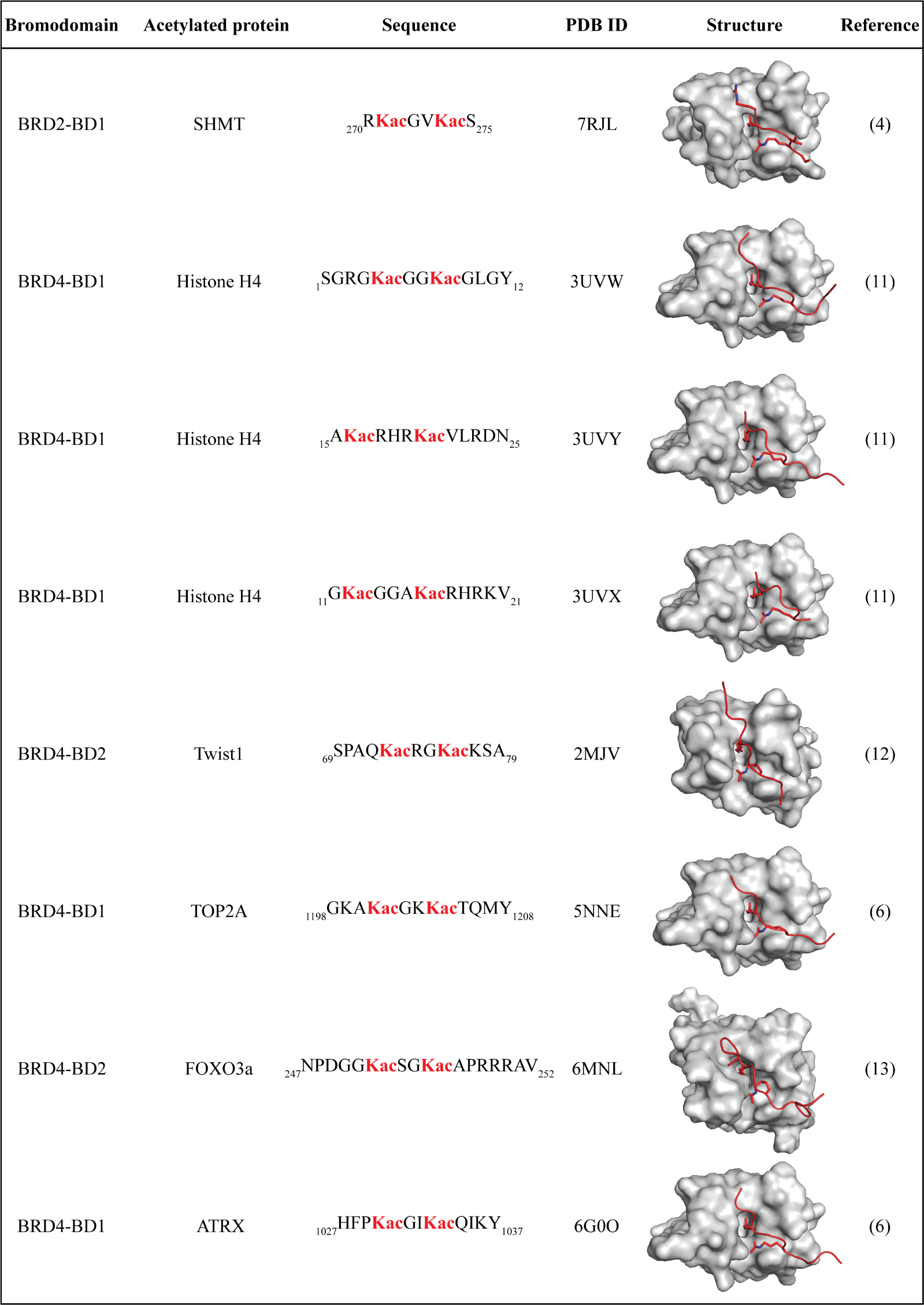
**

**
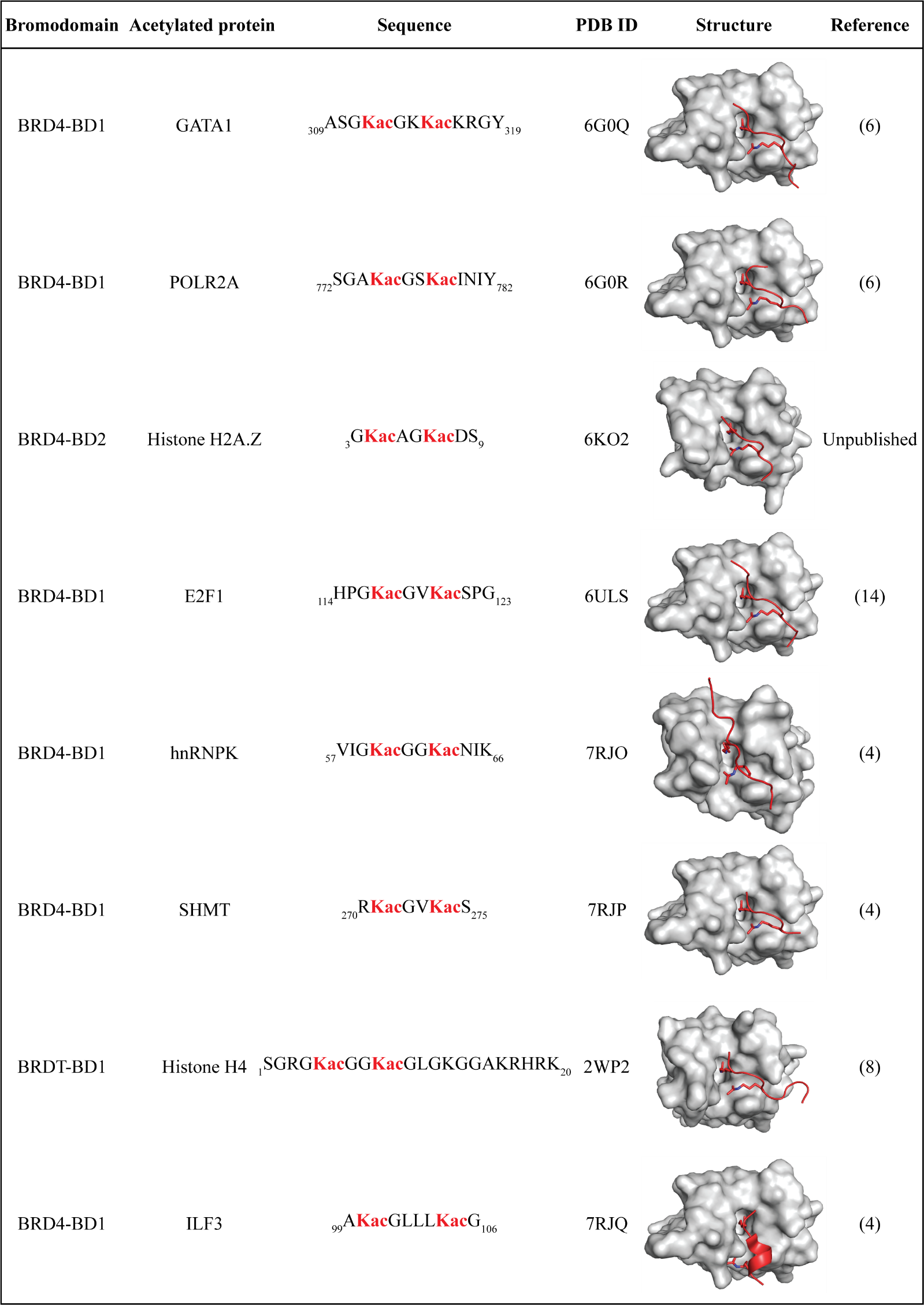

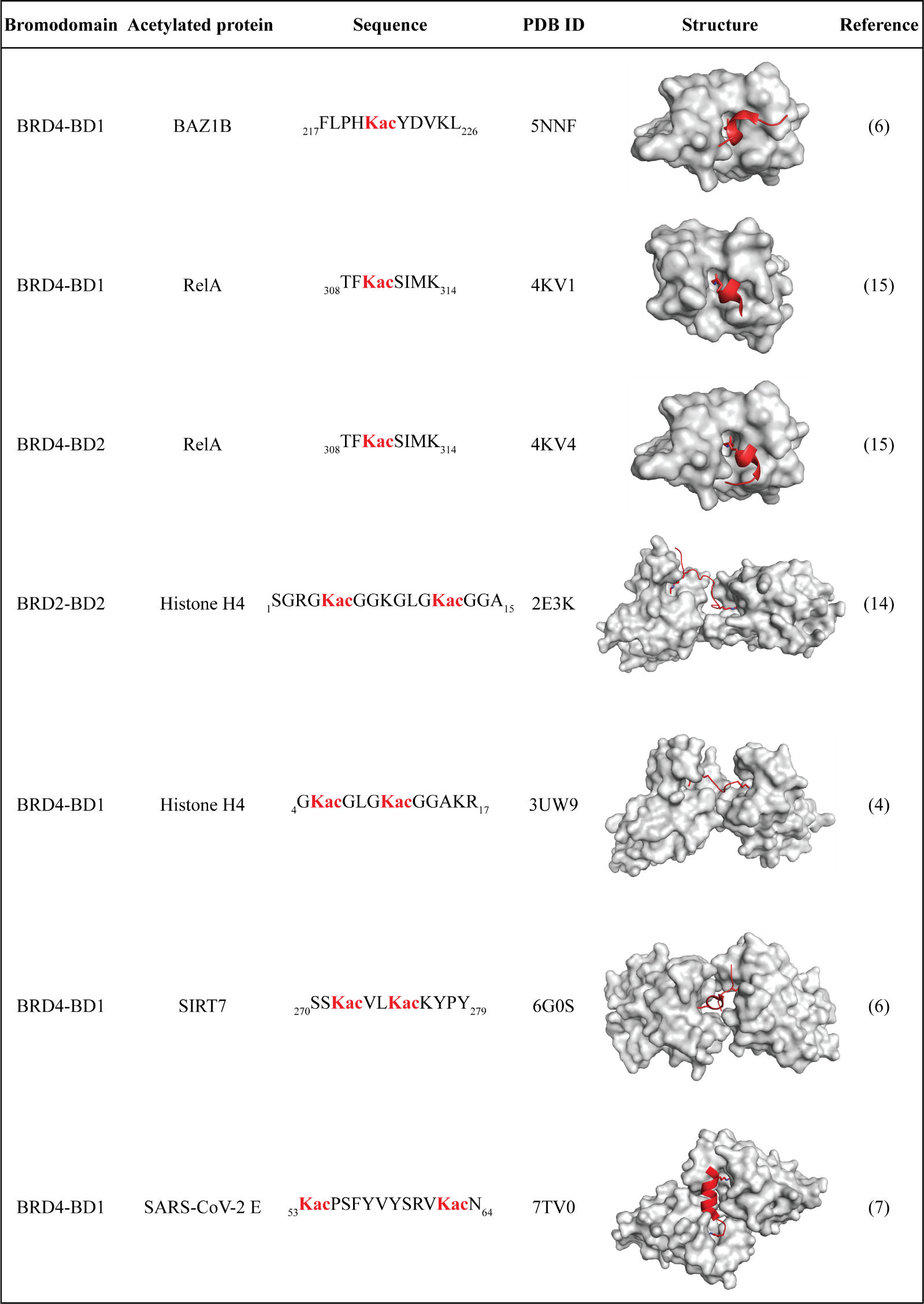
**

**FIGURES**


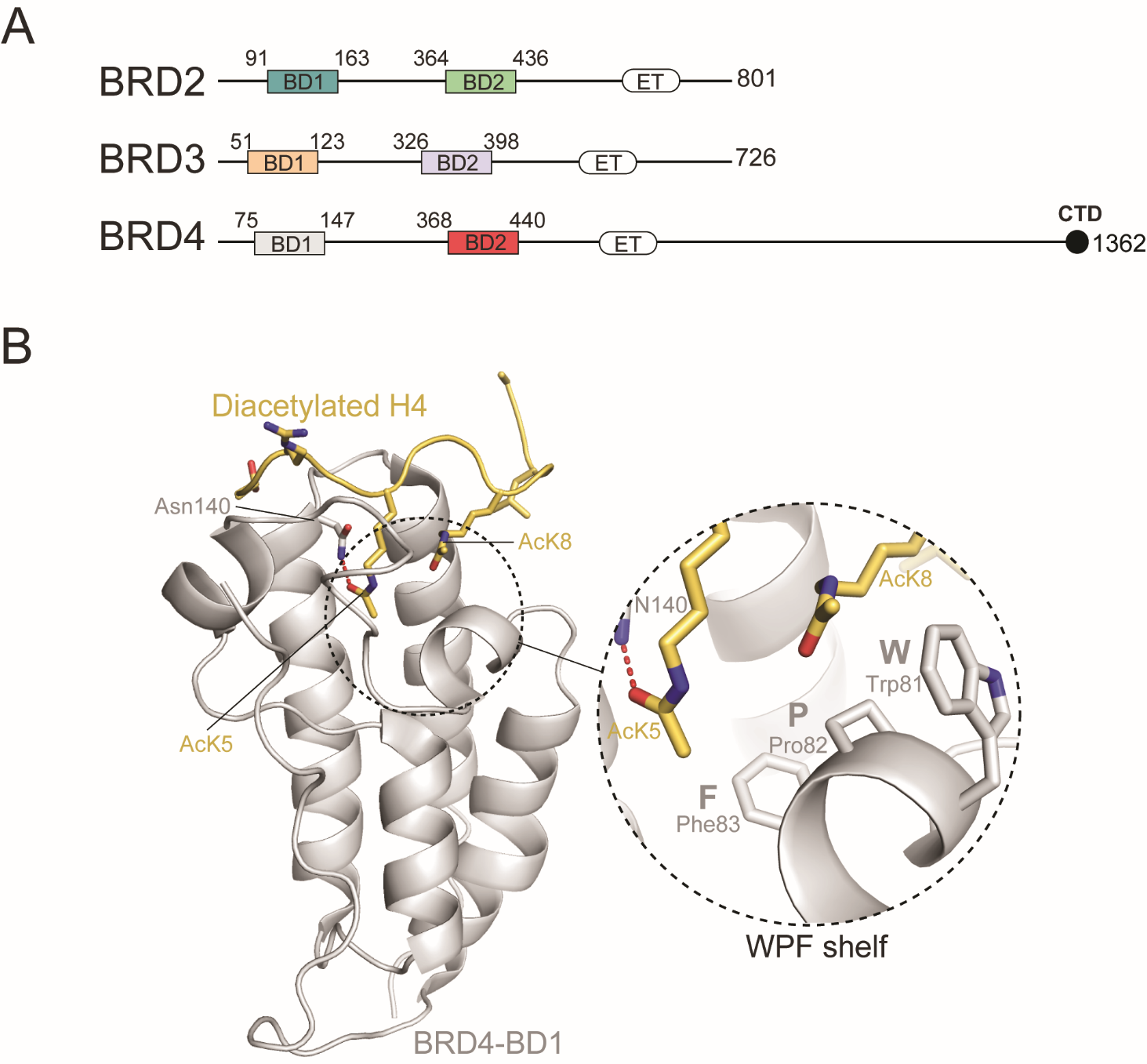


**Figure S1. The BET family of bromodomain containing proteins. A.** Topology diagrams of human BRD2, BRD3 and BRD4, showing the location of the BDs used in this study. BD stands for bromodomain, ET stands for extra-terminal domain, and CTD stands for *C*-terminal domain. **B.** The X-ray crystal structure of BRD4-BD1 (*grey*) in complex with a diacetylated histone H4 peptide (*yellow*, PDB ID: 3UVW). *Left*: Ribbon representation of the BRD4-BD1:H4 complex. The conserved asparagine (Asn140) that makes a hydrogen bond with the N-terminal AcK residue from the diacetylated histone H4 motif (AcK5) are displayed as sticks and the hydrogen bond formed between them is indicated by the *orange dashed line*. *Right inset:* A close-up of the WPF shelf that forms significant van der Waals interactions with C-terminal AcK (AcK8) of the diacetylated motif in histone H4 is shown in the diagram enclosed in the *dashed circle*.


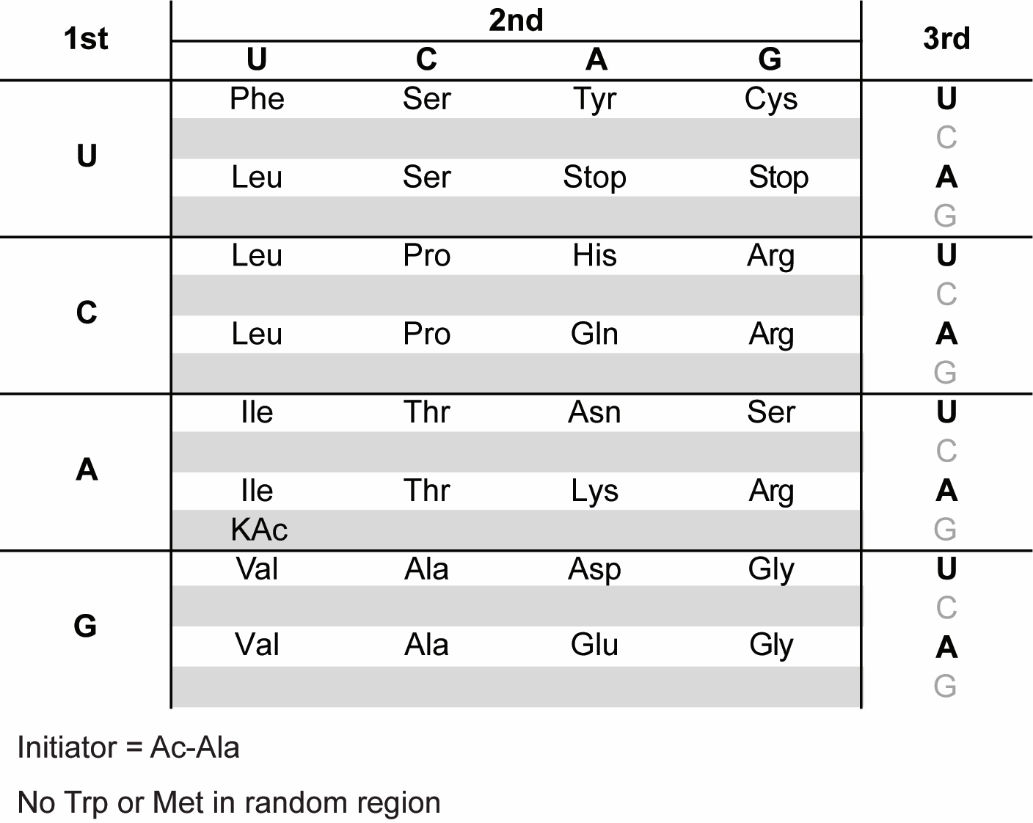


**Figure S2**. **Codon table for the RaPID screens showing amino acid assignment for each codon encoded in the randomised library.** Methionine was replaced by AcK and the initiator tRNA charged with *^N^*Ac-Ala.


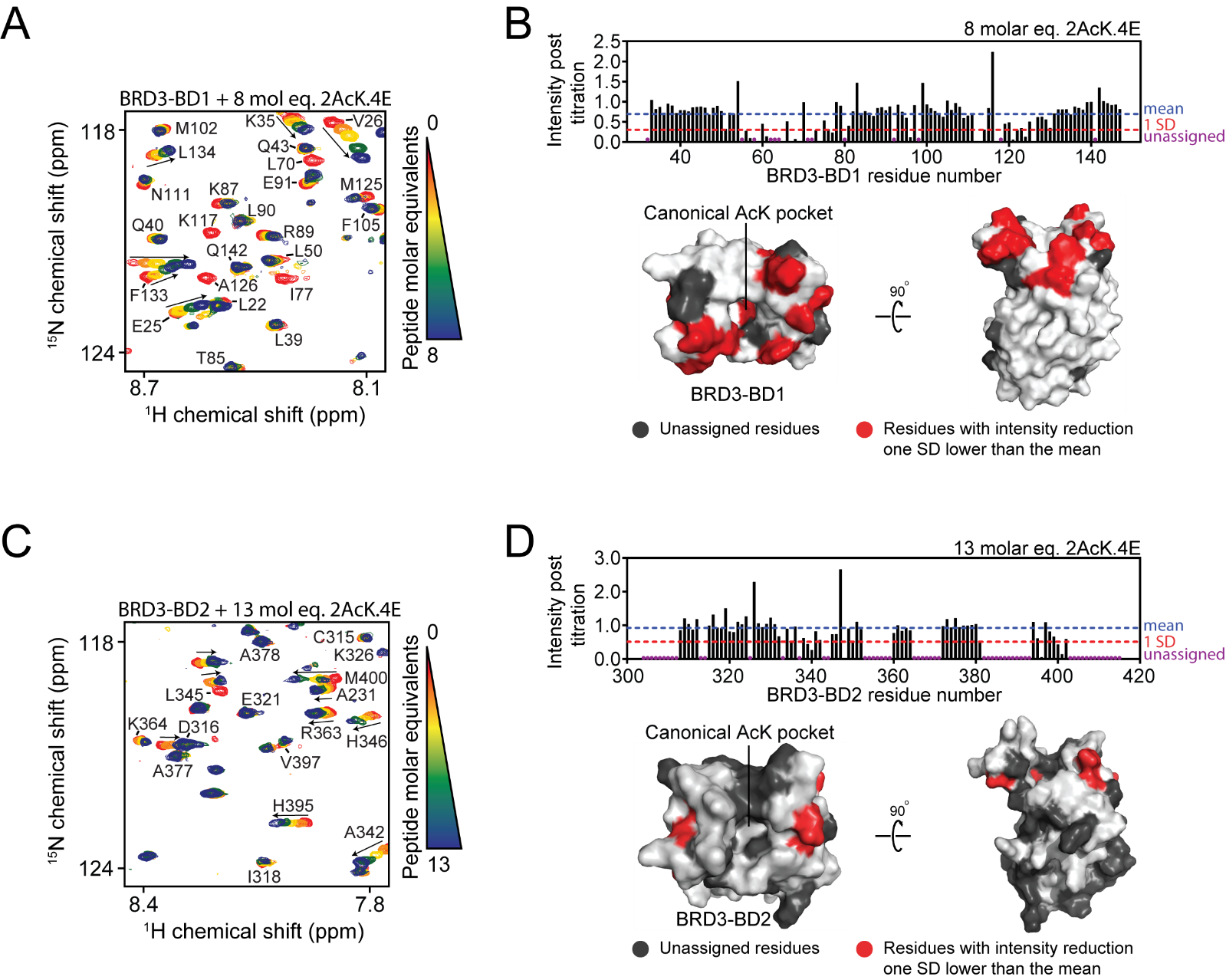


**Figure S3. ^15^N-HSQC titrations for 2AcK.4E into the BDs of BRD3 and structural mapping of the interactions.** For all ^15^N-HSQC titrations, BDs were used at a concentration of ~30 µM and the assignments and direction of movement of some signals are indicated. **A.** ^15^N-HSQC spectra of BRD3-BD1 alone (*red*) and in the presence of increasing concentrations of 2AcK.4E (up to 8 molar equivalents of the peptide, *blue*). **B.** *Top:* Quantitation of change in ^15^N-HSQC signal intensity for each signal following the addition of the indicated amount of 2AcK.4E to BRD3-BD1. The mean intensity across all signals in the titration is marked by the blue dashed line and the intensity reduction one standard deviation (1 SD) below the mean is indicated by the red dashed line. Unassigned residues in the titration are indicated by purple circles. *Bottom:* Residues for which signals are reduced in intensity by at least 1 SD from the mean reduction are mapped onto the structure of BRD3-BD1 (light grey) in red. Unassigned residues are coloured dark grey. **C.** ^15^N-HSQC spectra of BRD3-BD2 alone (*red*) and in the presence of increasing concentrations of 2AcK.4E (up to 13 molar equivalents of the peptide, *blue*). **D.** *Top:* Quantitation of change in ^15^N-HSQC signal intensity for each signal following the addition of the indicated amount of 2AcK.4E to BRD3-BD2. The mean intensity across all signals in the titration is marked by the blue dashed line and the intensity reduction one standard deviation (1 SD) below the mean is indicated by the red dashed line. Unassigned residues in the titration are indicated by purple circles. *Bottom:* Residues for which signals are reduced in intensity by at least 1 SD from the mean reduction are mapped onto the structure of BRD3-BD2 (light grey) in red. Unassigned residues are coloured dark grey.

**
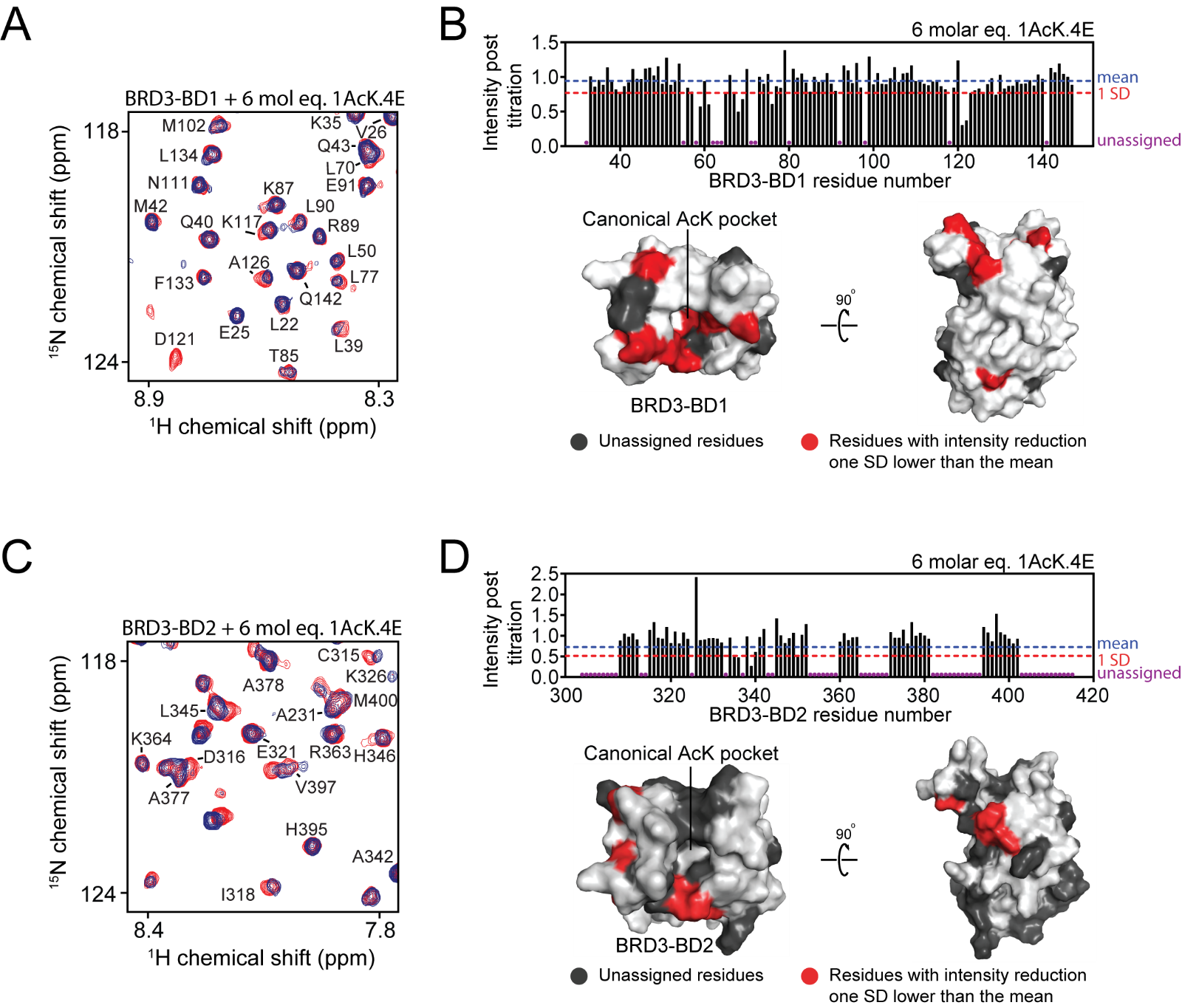
**

**Figure S4. ^15^N-HSQC titrations for 1AcK.4E into the BDs of BRD3 and structural mapping of the interactions.** For all ^15^N-HSQC titrations, BDs were used at a concentration of ~30 µM and the assignments of some signals are indicated. **A.** ^15^N-HSQC spectra of BRD3-BD1 alone (*red*) and in the presence of 6 molar equivalents of 1AcK.4E (*blue*). **B.** *Top:* Quantitation of change in ^15^N-HSQC signal intensity for each signal following the addition of the indicated amount of 1AcK.4E to BRD3-BD1. The mean intensity across all signals in the titration is marked by the blue dashed line and the intensity reduction one standard deviation (1 SD) below the mean is indicated by the red dashed line. Unassigned residues in the titration are indicated by purple circles. *Bottom:* Residues for which signals are reduced in intensity by at least 1 SD from the mean reduction are mapped onto the structure of BRD3-BD1 (light grey) in red. Unassigned residues are coloured dark grey. **C.** ^15^N-HSQC spectra of BRD3-BD2 alone (*red*) and in the presence of 6 molar equivalents of 1AcK.4E (*blue*). **D.** *Top:* Quantitation of change in ^15^N-HSQC signal intensity for each signal following the addition of the indicated amount of 1AcK.4E to BRD3-BD2. The mean intensity across all signals in the titration is marked by the blue dashed line and the intensity reduction one standard deviation (1 SD) below the mean is indicated by the red dashed line. Unassigned residues in the titration are indicated by purple circles. *Bottom* Residues for which signals are reduced in intensity by at least 1 SD from the mean reduction are mapped onto the structure of BRD3-BD2 (light grey) in red. Unassigned residues are coloured dark grey.


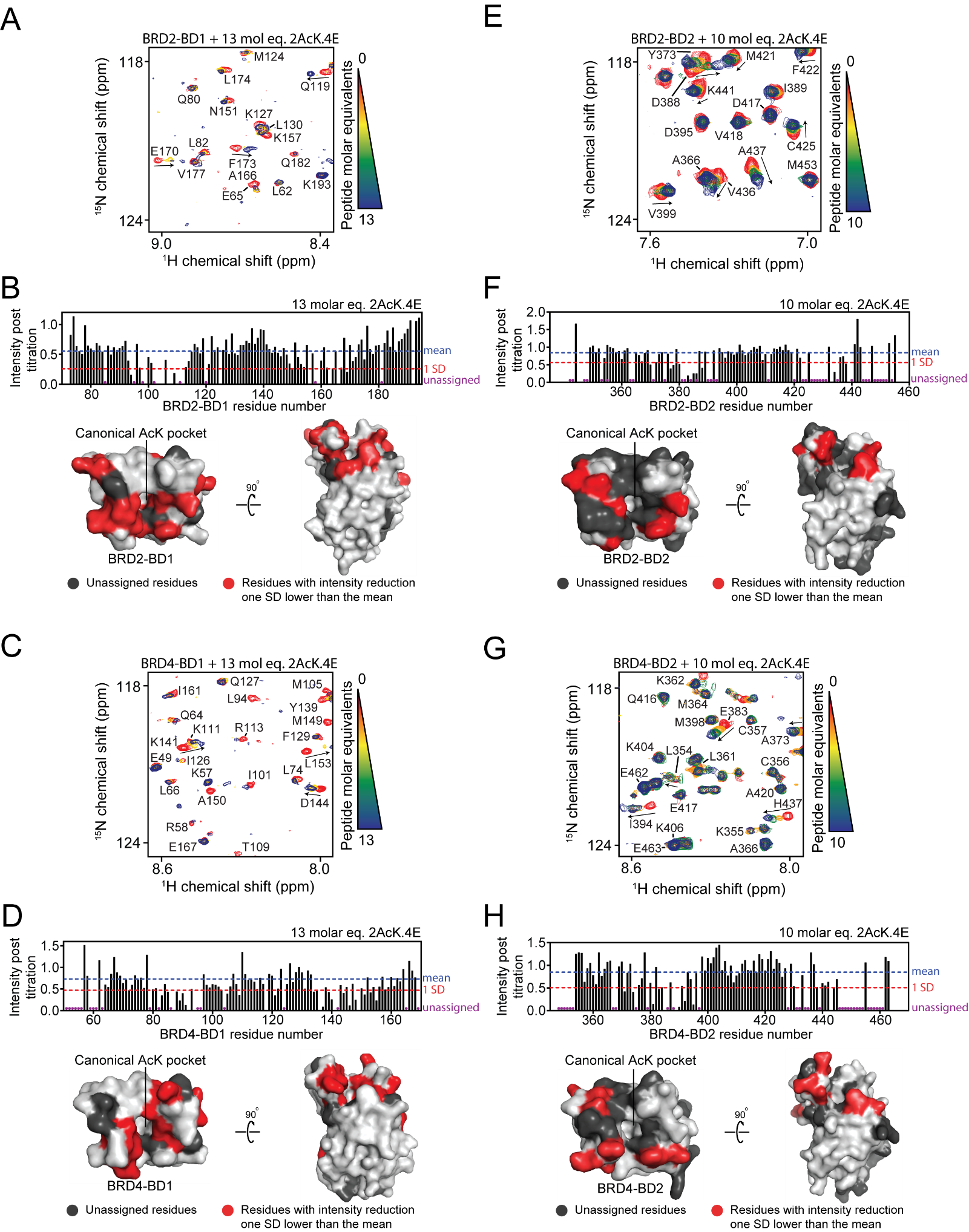


**Figure S5. Peptide 2AcK.4E binds the BDs from BRD2 and BRD4 at the canonical binding site.** For all ^15^N-HSQC titrations, BDs were used at a concentration of ~30 µM and the assignments and direction of movement of some signals are indicated. **A.** ^15^N-HSQC spectra of BRD2-BD1 alone (*red*) and in the presence of increasing concentrations of 2AcK.4E (up to 13 molar equivalents of the peptide, *blue*). **B.** *Top:* Quantitation of change in ^15^N-HSQC signal intensity for each signal following the addition of the indicated amount of 2AcK.4E to BRD2-BD1. The mean intensity across all signals in the titration is marked by the blue dashed line and the intensity reduction one standard deviation (1 SD) below the mean is indicated by the red dashed line. Unassigned residues in the titration are indicated by purple circles. *Bottom:* Residues for which signals are reduced in intensity by at least 1 SD from the mean reduction are mapped onto the structure of BRD2-BD1 (light grey) in red. Unassigned residues are coloured dark grey. **C.** ^15^N-HSQC spectra of BRD4-BD1 alone (*red*) and in the presence of increasing concentrations of 2AcK.4E (up to 13 molar equivalents of the peptide, *blue*). **D.** *Top:* Quantitation of change in ^15^N-HSQC signal intensity for each signal following the addition of the indicated amount of 2AcK.4E to BRD4-BD1. The mean intensity across all signals in the titration is marked by the blue dashed line and the intensity reduction one standard deviation (1 SD) below the mean is indicated by the red dashed line. Unassigned residues in the titration are indicated by purple circles. *Bottom:* Residues for which signals are reduced in intensity by at least 1 SD from the mean reduction are mapped onto the structure of BRD4-BD1 (light grey) in red. Unassigned residues are coloured dark grey. **E.** ^15^N-HSQC spectra of BRD2-BD2 alone (*red*) and in the presence of increasing concentrations of 2AcK.4E (up to 10 molar equivalents of the peptide, *blue*). **F.** *Top:* Quantitation of change in ^15^N-HSQC signal intensity for each signal following the addition of the indicated amount of 2AcK.4E to BRD2-BD2. The mean intensity across all signals in the titration is marked by the blue dashed line and the intensity reduction one standard deviation (1 SD) below the mean is indicated by the red dashed line. Unassigned residues in the titration are indicated by purple circles. *Bottom:* Residues for which signals are reduced in intensity by at least 1 SD from the mean reduction are mapped onto the structure of BRD2-BD2 (light grey) in red. Unassigned residues are coloured dark grey. **G.** ^15^N-HSQC spectra of BRD4-BD2 alone (*red*) and in the presence of increasing concentrations of 2AcK.4E (up to 10 molar equivalents of the peptide, *blue*). **H.** *Top:* Quantitation of change in ^15^N-HSQC signal intensity for each signal following the addition of the indicated amount of 2AcK.4E to BRD4-BD2. The mean intensity across all signals in the titration is marked by the blue dashed line and the intensity reduction one standard deviation (1 SD) below the mean is indicated by the red dashed line. Unassigned residues in the titration are indicated by purple circles. *Bottom:* Residues for which signals are reduced in intensity by at least 1 SD from the mean reduction are mapped onto the structure of BRD4-BD2 (light grey) in red. Unassigned residues are coloured dark grey.

**
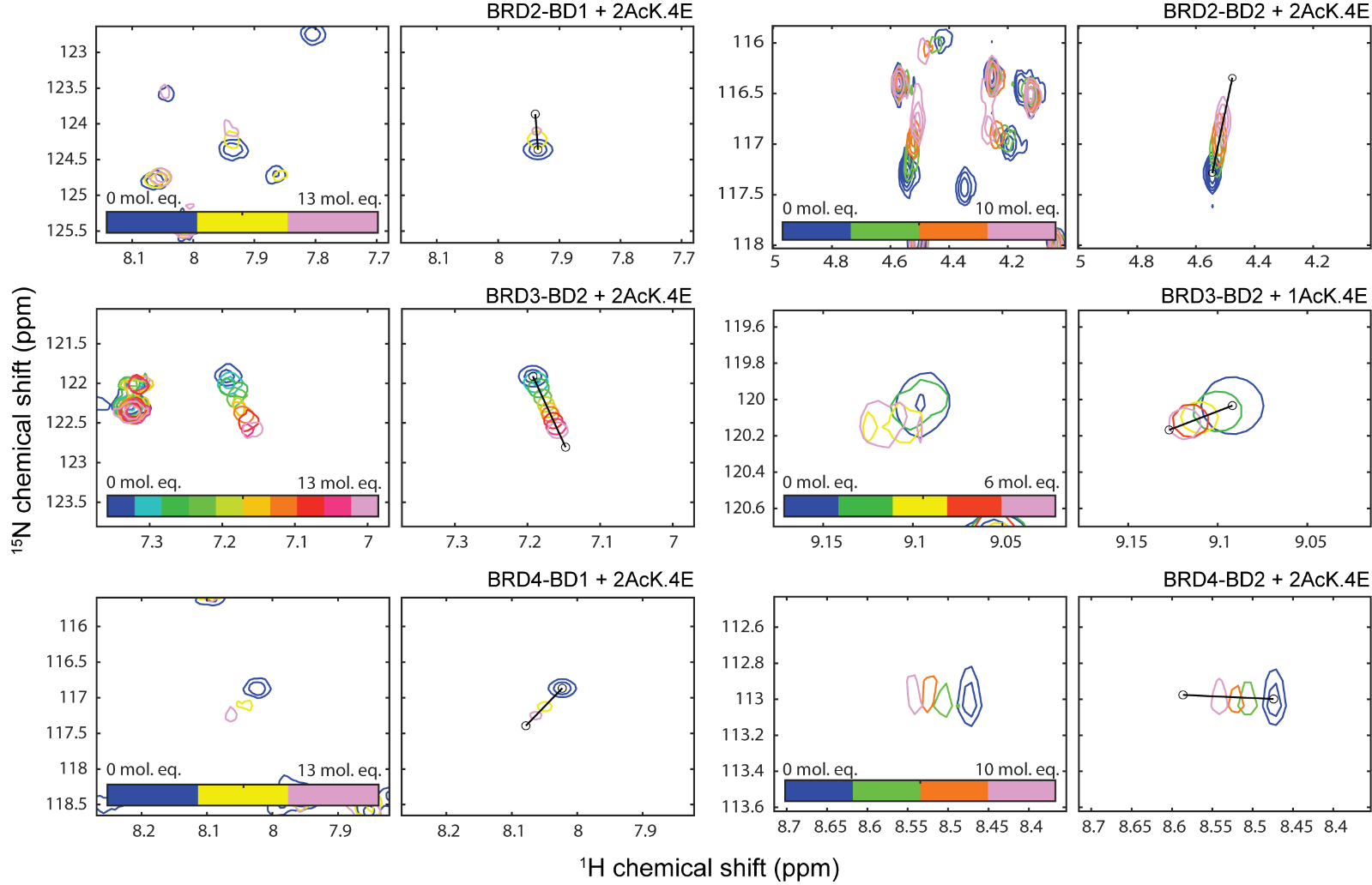
**

**Figure S6. TITAN fits to ^15^N-HSQC titration data for the indicated titrations.** In each case, experimental data for a representative peak are shown on the left and full lineshape fits for the same peak from TITAN are shown on the right. Circles indicate the predicted start and finish positions for the corresponding signal. Titrations were carried out once and fits were calculated to at least two peaks in each titration.


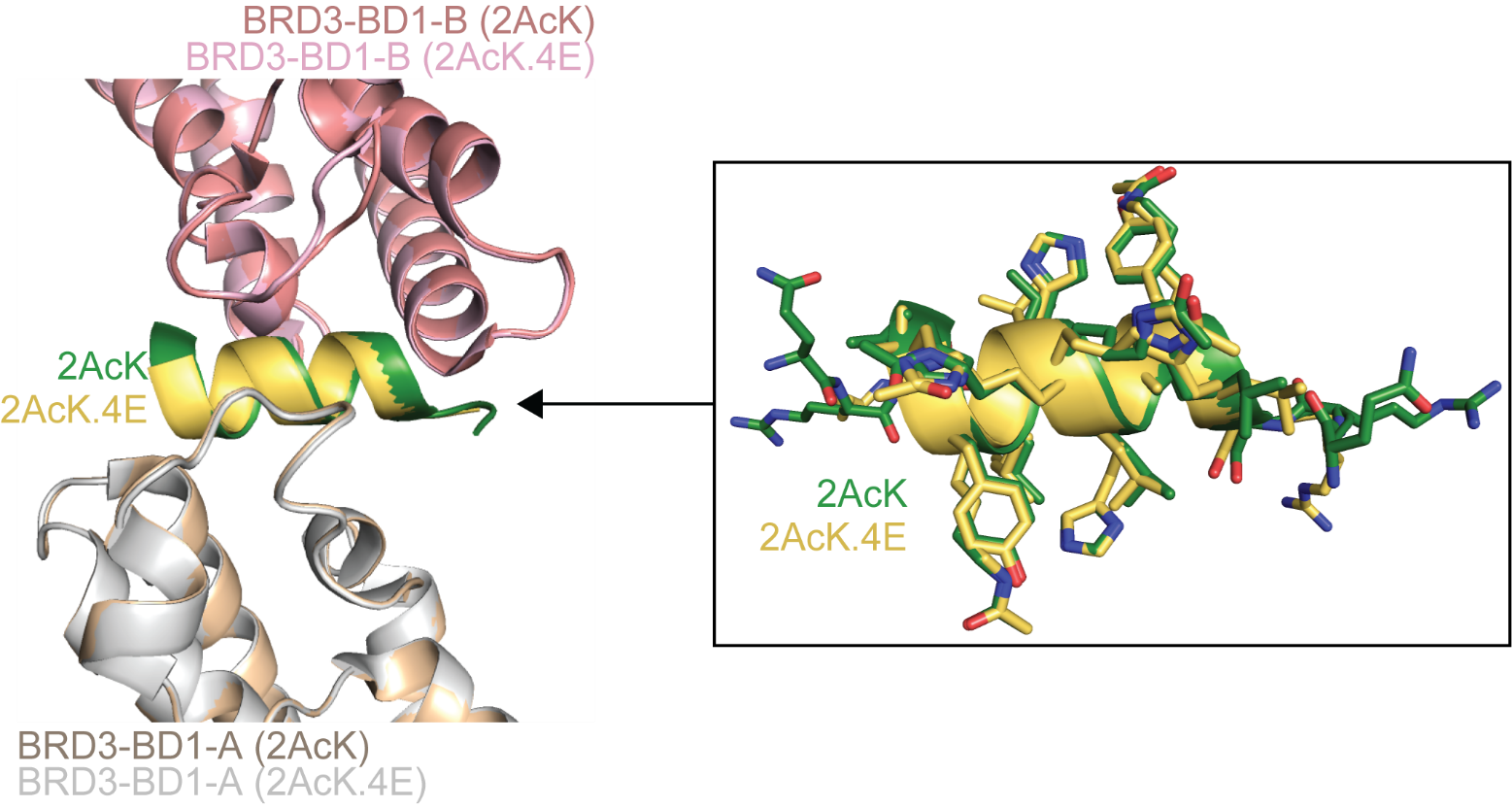


**Figure S7. Superposition of the structures of BRD3-BD1 in complex with 2AcK (PDB ID: 7TO8) and 2AcK.4E (PDB ID: 7TO9).** The *right panel* shows a section of the superposition of the two structures. The BDs and peptides from each structure are labelled and shown in different colours. The *left panel* shows a closeup of the superposition of 2AcK and 2AcK.4E. The dual sidechain occupancies of both peptides are displayed in the inset. The sidechains and backbones of both peptides occupy essentially the same positions; however, more of the *N*- and *C*-terminal tails of 2AcK (*green*) visible in the structure than 2AcK.4E (*yellow*).


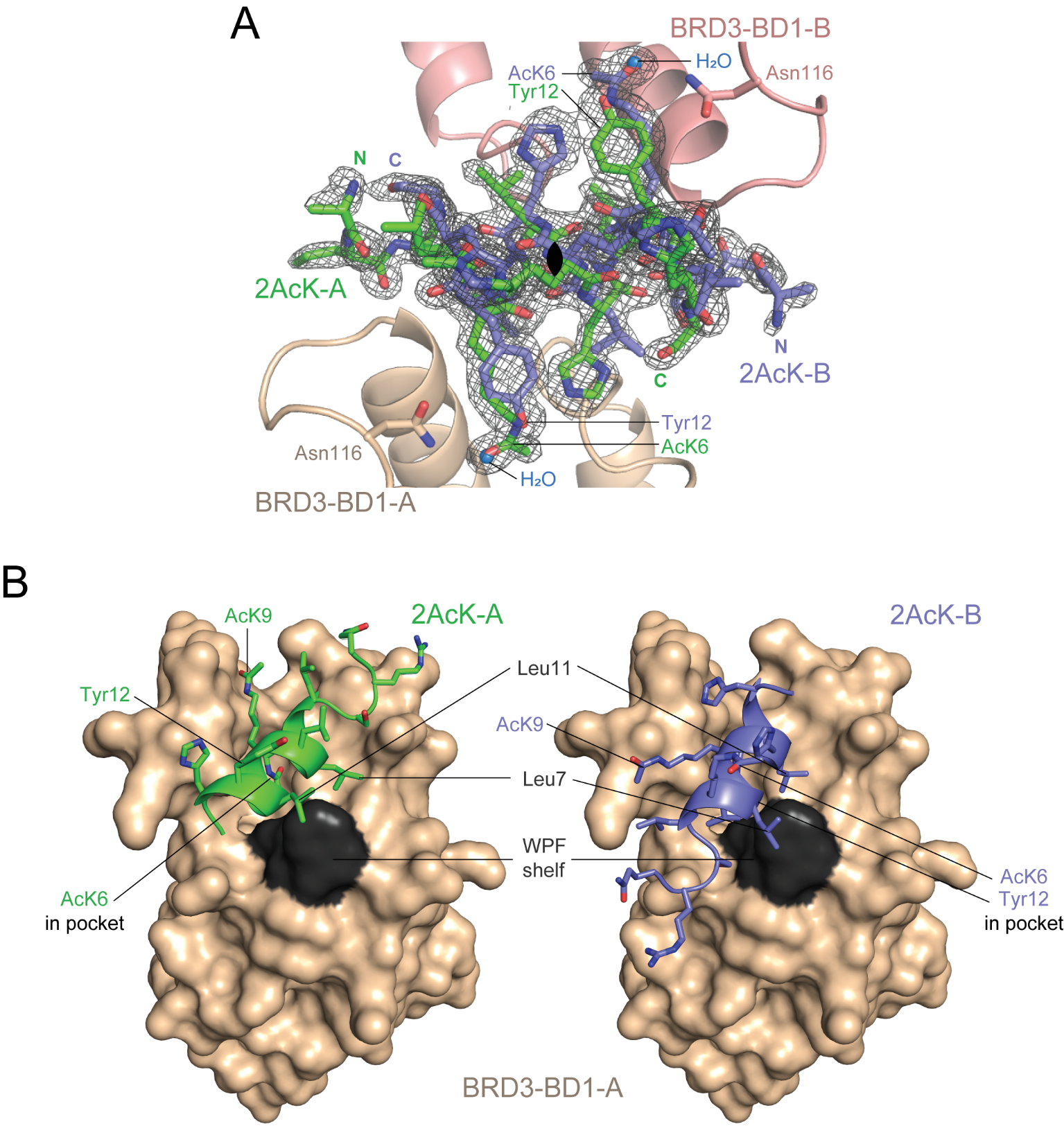


**Figure S8. Structure of BRD3-BD1 bound to 2AcK. A.** Electron density (2F_O_ – F_C_) observed in the space between the two copies of BRD3-BD1 in the asymmetric unit. The density (shown as a mesh) is clearly better fitted by using a model that incorporates two copies of the peptide (each at 50% occupancy, *green* and *purple*) rotated 180° about the indicated axis (*black ellipse*). The pseudo-two-fold axis of rotation, which maps each BD onto the position of the other and approximately maps the overall helical shape of the peptide backbone onto itself (but running in the opposite direction) is indicated with a *black* *ellipse*. It can be seen that AcK6 and Tyr12 of 2AcK occupy the AcK-binding pocket of BRD3-BD1-A and BRD3-BD1-B, respectively. **B.** Comparison of peptide binding geometry for binding to the two BDs in the asymmetric unit. The orientation of peptide 2AcK-B that binds BRD3-BD1-B using Tyr12 is superimposed onto BRD3-BD1-A to demonstrate the relative orientations of 2AcK. The WPF shelf is indicated in *black*.

**
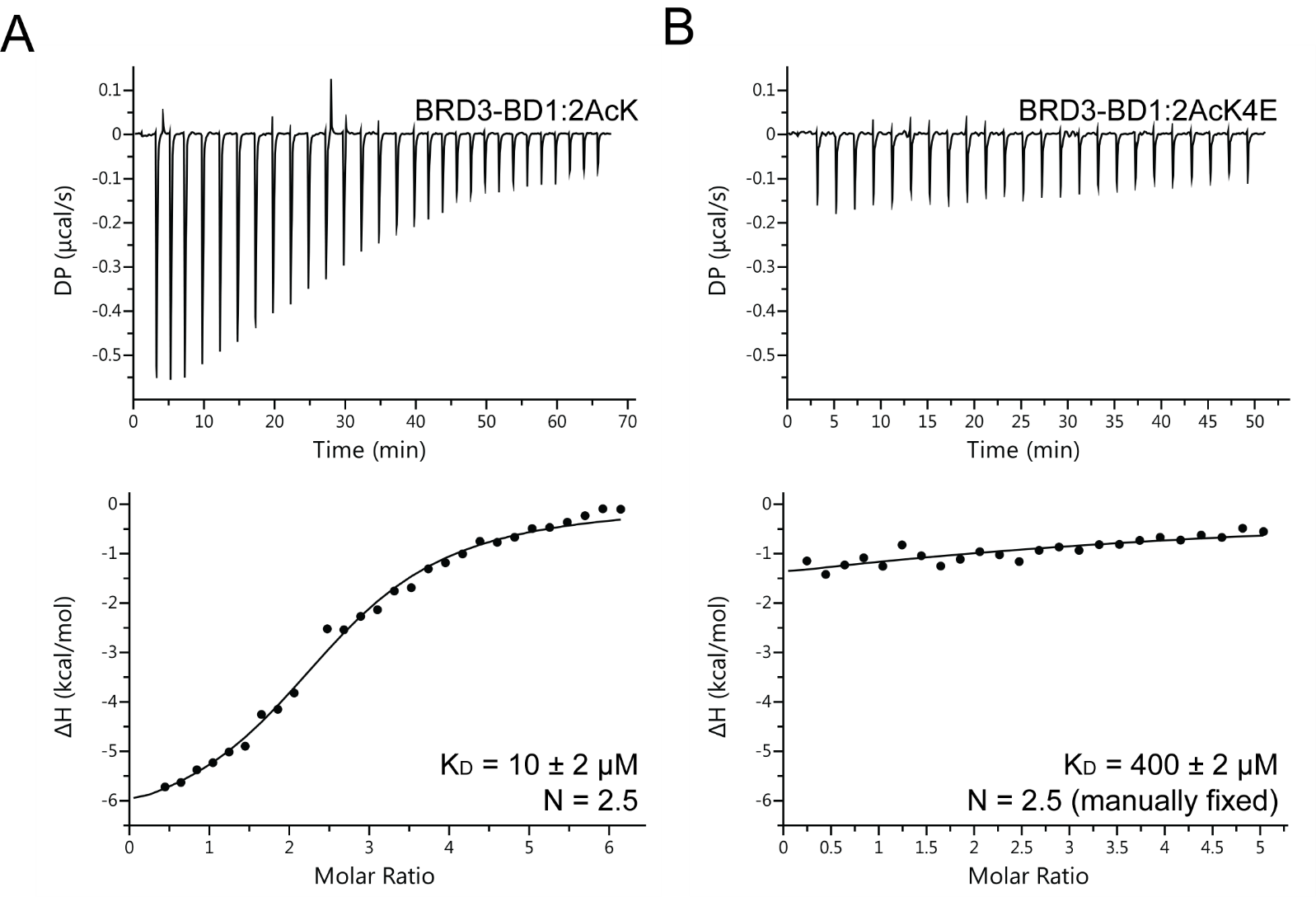
Figure S9. ITC analysis of the interaction between BRD3-BD1 and the 2AcK peptides. A.** ITC data for titration of BRD3-BD1 into 2AcK. *Top*: Exothermic enthalpy ITC curve for the titration of BRD3-BD1 into 2AcK. *Bottom*: Fit of the ITC data to a binding isotherm. The derived *K_D_* and N value for the interaction are shown. **B.** ITC data for titration of BRD3-BD1 into 2AcK.4E. *Top*: Exothermic enthalpy ITC curve for the titration of BRD3-BD1 into 2AcK.4E. *Bottom*: Fits of the ITC data to a binding isotherm. The derived *K_D_* is shown (N was fixed to 2.5 in the fitting procedure).

K_D_ = 300 ± 2 μM


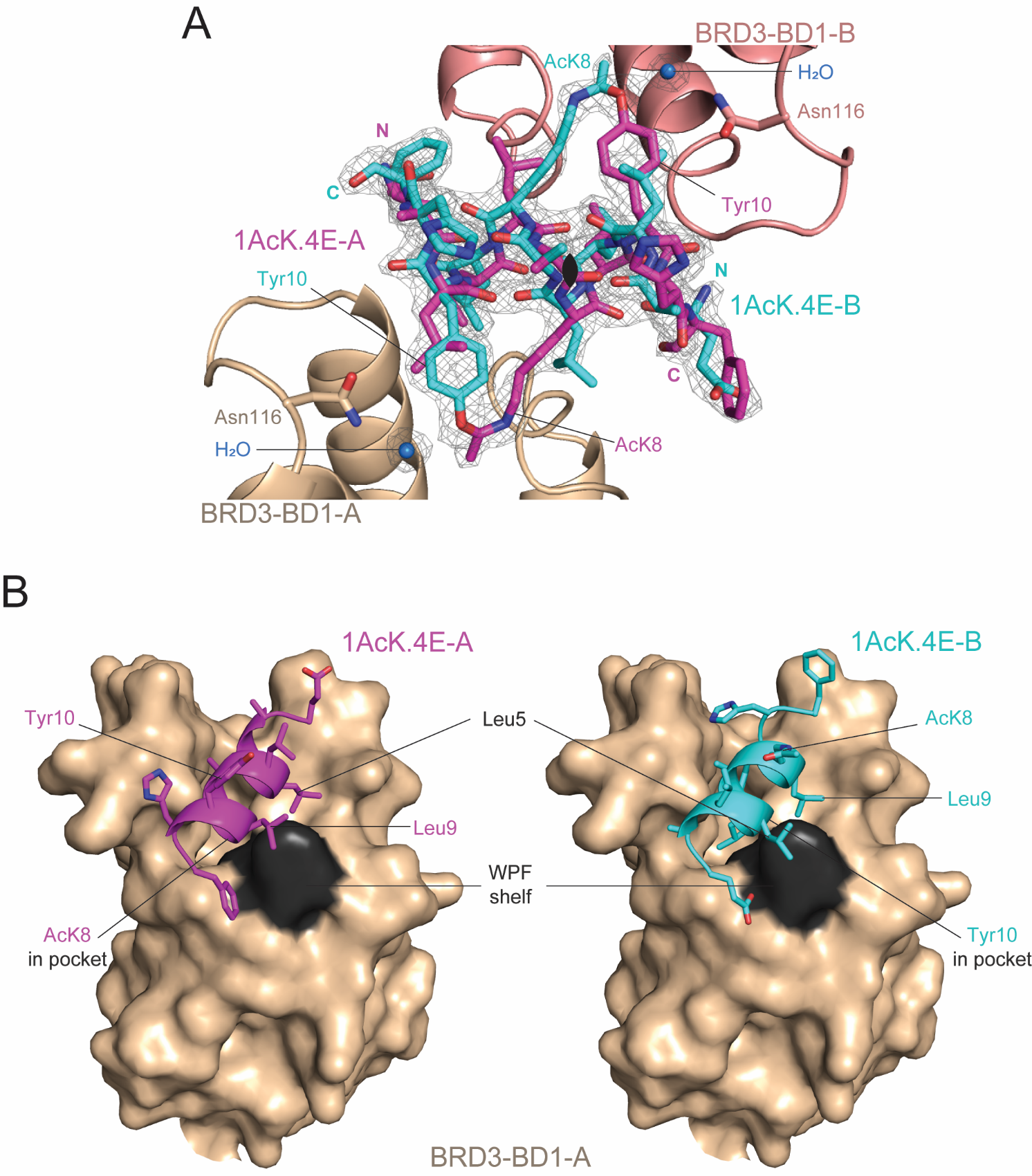


**Figure S10. Structure of BRD3-BD1 bound to 1AcK.4E. A.** Electron density (2F_0_ – F_c_) observed in between the two copies of BRD3-BD1 in the asymmetric unit. The density (shown as a mesh) is clearly better fitted by using a model that incorporates two copies of the peptide (each at 50% occupancy, *magenta* and *cyan*) rotated 180° about the indicated axis (*black ellipse*). The pseudo-two-fold axis of rotation, which maps each BD onto the position of the other and approximately maps the overall helical shape of the peptide backbone onto itself (but running in the opposite direction) is indicated with a *black* *ellipse*. It can be seen that AcK8 and Tyr10 of 1AcK.4E occupy the AcK-binding pocket of BRD3-BD1-A and BRD3-BD1-B, respectively. **B.** Comparison of peptide binding geometry for binding to the two BDs in the asymmetric unit. The orientation of peptide 1AcK.4E-B that binds BRD3-BD1-B using Tyr10 is superimposed onto BRD3-BD1-A to demonstrate the relative orientations of 1AcK.4E.


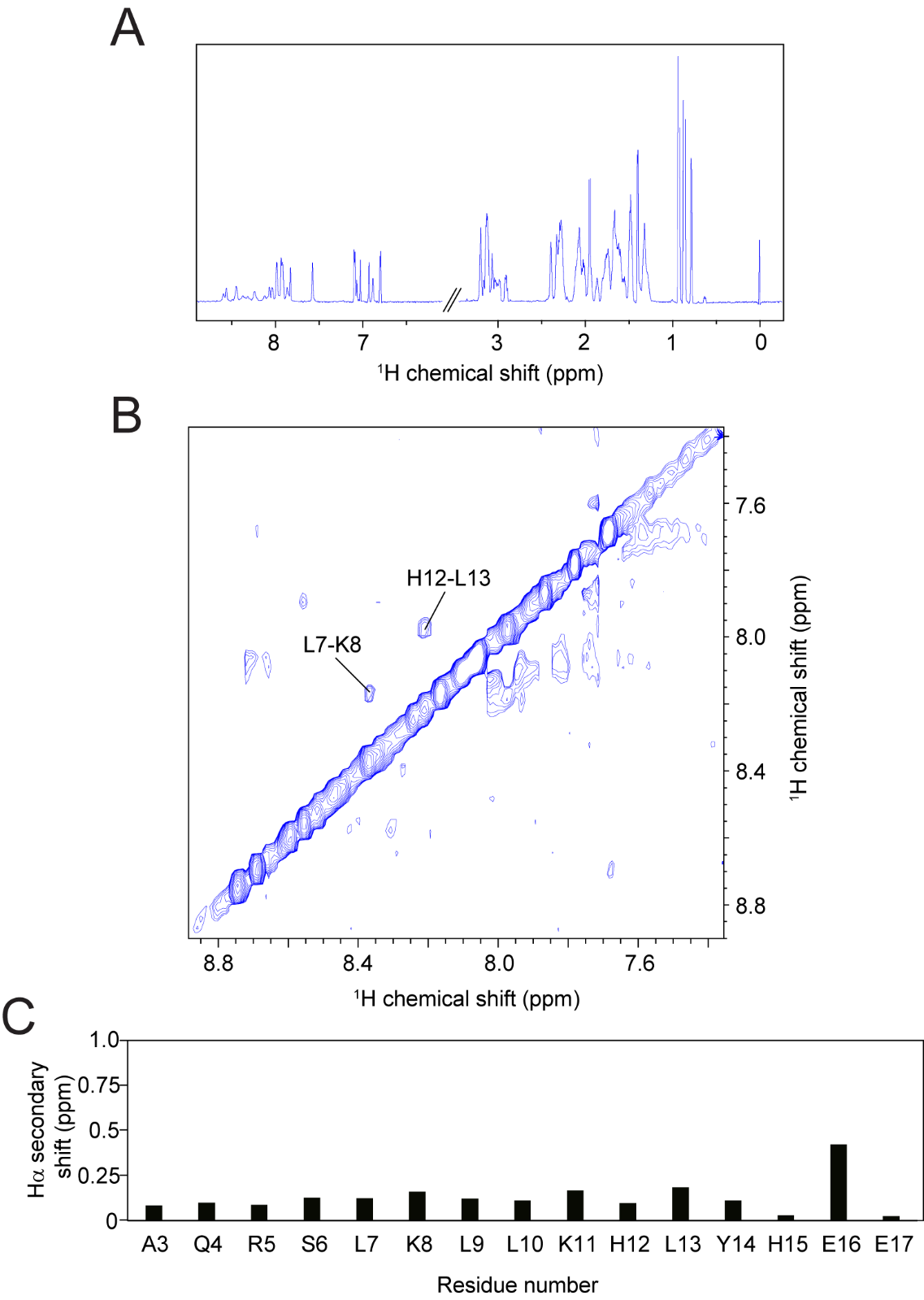


**Figure S11. 2AcK.4E is intrinsically disordered. A.** Portions of a 1D ^1^H NMR spectrum of 2AcK.4E (recorded at 800 MHz, 25 °C). **B**. HN-HN region of a 2D NOESY spectrum of 2AcK.4E (mixing time = 100 ms). The lack of a significant number of NOEs connecting pairs of HN protons strongly suggests that the peptide does not form appreciable amounts of α-helix. **C**. Secondary chemical shifts for Hα protons of 2AcK.4E, obtained by subtracting the random coil chemical shift for each residue from the observed chemical shift. Calculations were performed on the CSI 3.0 server of the Wishart lab (<http://csi3.wishartlab.com/>). The small and uniformly positive nature of the secondary shifts is consistent with a largely disordered polypeptide with a weak preference for an α-helical conformation.

**
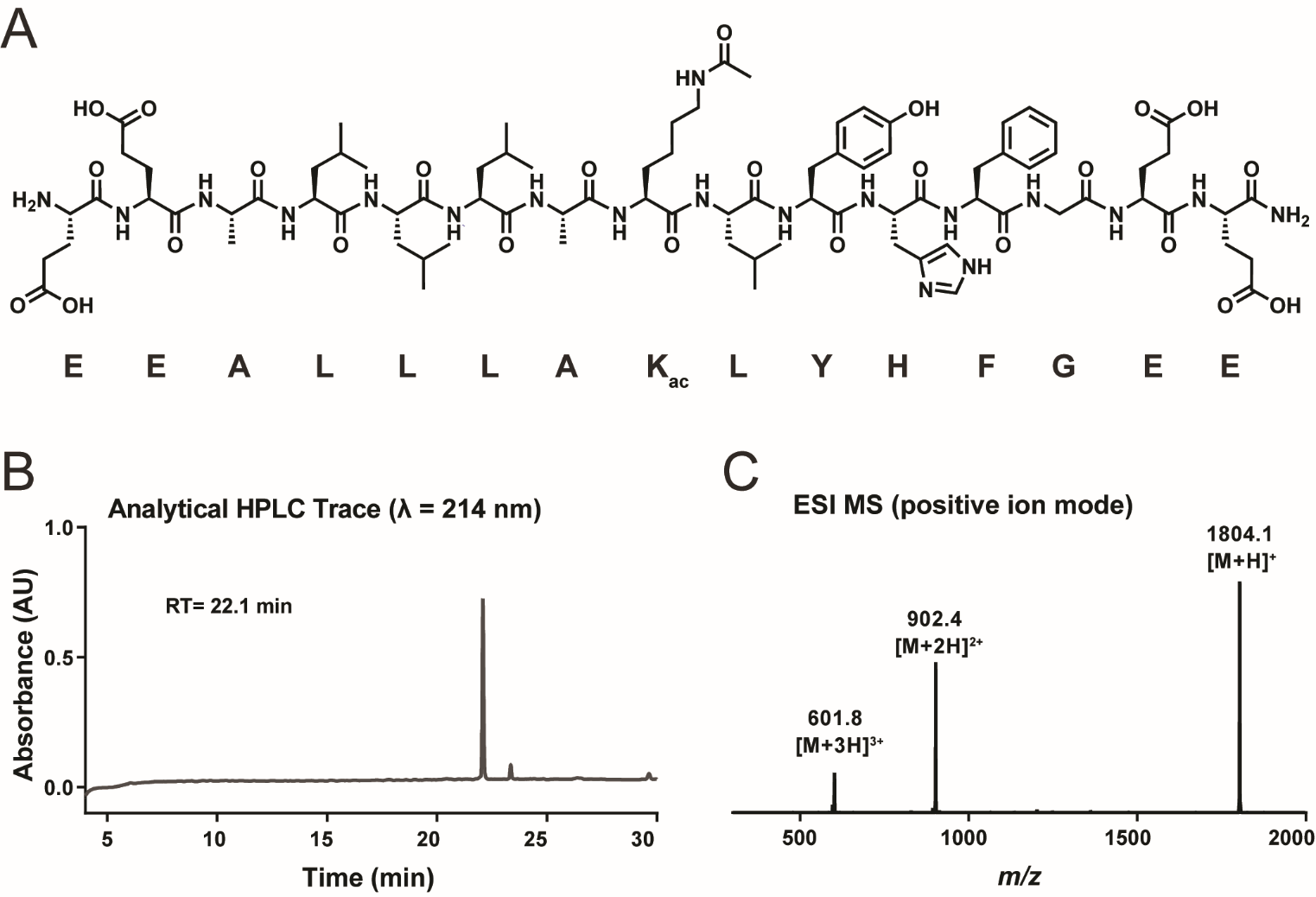
**

**Figure S12. HPLC trace and MS spectrum for peptide 1AcK.4E. A.** Structure and sequence of peptide 1AcK.4E. **B.** Analytical HPLC chromatogram. R_t_ = 22.1 min (1 to 60 vol.% MeCN in H_2_O with 0.1 vol.% TFA over 30 min, λ = 214 nm). **C.** Low resolution peptide mass spectrum in positive ion ESI mode. Observed masses at *m/z* 1804.1 [M+H]^+^, 902.4 [M+2H]^2+^, 601.8 [M+3H]^3+^.


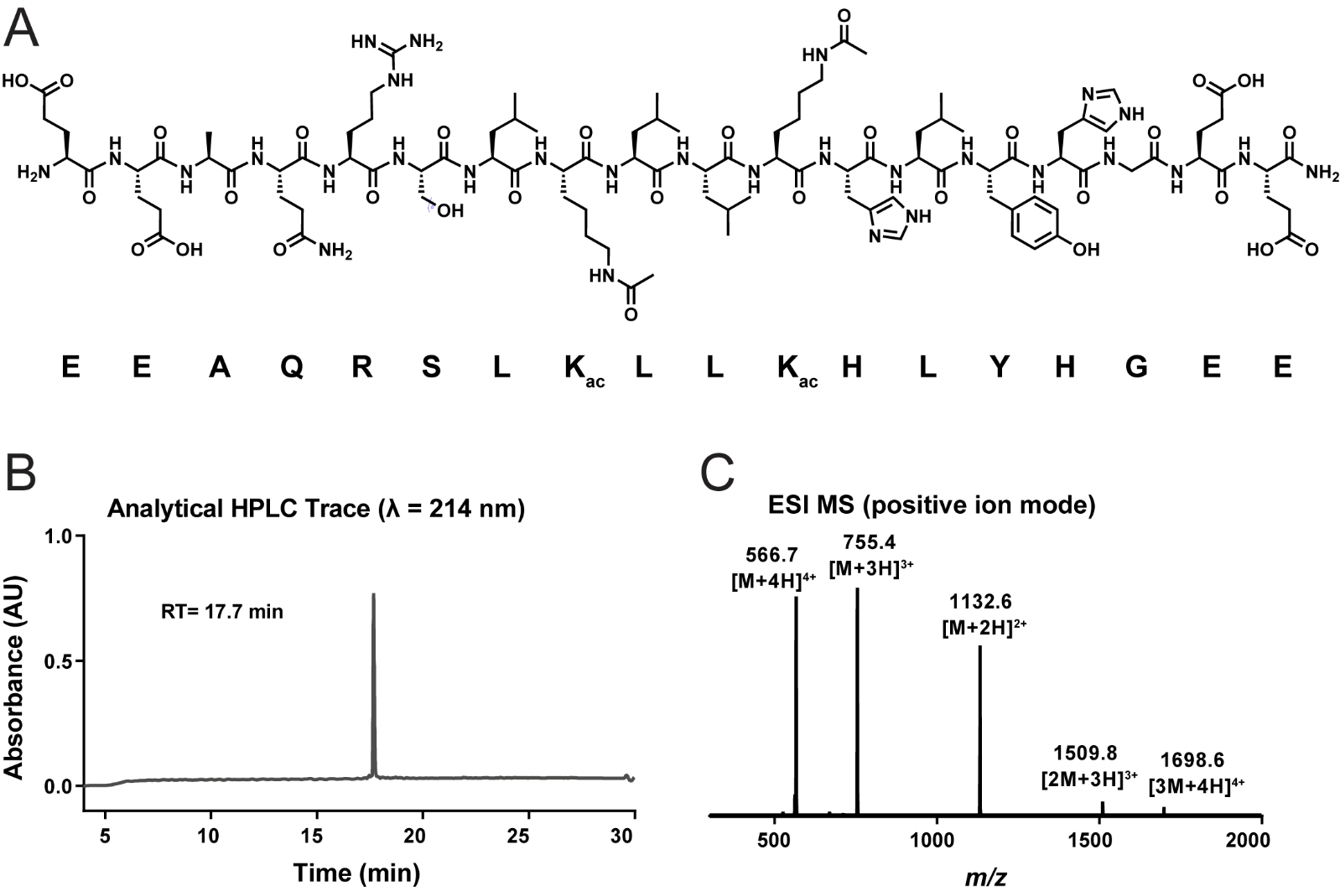


**Figure S13. HPLC trace and MS spectrum for peptide 2AcK.4E. A.** Structure and sequence of peptide 2AcK.4E. **B.** Analytical HPLC chromatogram. R_t_ = 17.7 min (1 to 60 vol.% MeCN in H_2_O with 0.1 vol.% TFA over 30 min, λ = 214 nm). **C.** Low resolution peptide mass spectrum in positive ion ESI mode. Observed masses at *m/z* 1698.6 [3M+4H]^4+^, 1509.8 [2M+3H]^3+^, 1132.6 [M+2H]^2+^, 755.4 [M+3H]^3+^, 566.7 [M+4H]^4+^.

(1) 2DVQ, 2DVR, 2DVS

(2) 5U5S

(3) 5HJC

(4) 7RJM, 7RJN

(5) 3JVK

(6) 5NNC, 5NNG

(7) 7TUQ

(8)

(9)

(10)

(11)

(12)

(13)

(14)

(15)

**REFERENCES**

1. Umehara, T., Nakamura, Y., Jang, M. K., Nakano, K., Tanaka, A., Ozato, K., Padmanabhan, B., and Yokoyama, S. (2010) Structural basis for acetylated histone H4 recognition by the human BRD2 bromodomain. *J. Biol. Chem.* **285**, 7610–7618

2. Cheung, K. L., Zhang, F., Jaganathan, A., Sharma, R., Zhang, Q., Konuma, T., Shen, T., Lee, J. Y., Ren, C., Chen, C. H., Lu, G., Olson, M. R., Zhang, W., Kaplan, M. H., Littman, D. R., Walsh, M. J., Xiong, H., Zeng, L., and Zhou, M. M. (2017) Distinct Roles of Brd2 and Brd4 in Potentiating the Transcriptional Program for Th17 Cell Differentiation. *Mol. Cell*. **65**, 1068-1080.e5

3. Li, Y., Sabari, B. R., Panchenko, T., Wen, H., Zhao, D., Guan, H., Wan, L., Huang, H., Tang, Z., Zhao, Y., Roeder, R. G., Shi, X., Allis, C. D., and Li, H. (2016) Molecular Coupling of Histone Crotonylation and Active Transcription by AF9 YEATS Domain. *Mol. Cell*. **62**, 181–193

4. Wagner, S., Fedorov, E., Sudhamalla, B., Jnawali, H. N., Debiec, R., Ghosh, A., and Islam, K. (2021) Uncovering the Bromodomain Interactome using Site-Specific Azide-Acetyllysine Photochemistry, Proteomic Profiling and Structural Characterization. *bioRxiv*. 10.1101/2021.07.28.453719

5. Vollmuth, F., Blankenfeldt, W., and Geyer, M. (2009) Structures of the dual bromodomains of the P-TEFb-activating protein Brd4 at atomic resolution. *J. Biol. Chem.* **284**, 36547–36556

6. Lambert, J.-P., Picaud, S., Fujisawa, T., Hou, H., Savitsky, P., Uusküla-Reimand, L., Gupta, G. D., Abdouni, H., Lin, Z. Y., Tucholska, M., Knight, J. D. R., Gonzalez-Badillo, B., St-Denis, N., Newman, J. A., Stucki, M., Pelletier, L., Bandeira, N., Wilson, M. D., Filippakopoulos, P., and Gingras, A.-C. C. (2019) Interactome Rewiring Following Pharmacological Targeting of BET Bromodomains. *Mol. Cell*. **73**, 621–638

7. Vann, K. R., Acharya, A., Jang, S. M., Lachance, C., Zandian, M., Holt, T. A., Smith, A. L., Pandey, K., Durden, D. L., El-Gamal, D., Côté, J., Byrareddy, S. N., and Kutateladze, T. G. (2022) Binding of the SARS-CoV-2 envelope E protein to human BRD4 is essential for infection. *Structure*. **30**, 1224-1232.e5

8. Morinière, J., Rousseaux, S., Steuerwald, U., Soler-López, M., Curtet, S., Vitte, A.-L. L., Govin, J. J., Gaucher, J., Sadoul, K., Hart, D. J., Krijgsveld, J., Khochbin, S., Müller, C. W., and Petosa, C. (2009) Cooperative binding of two acetylation marks on a histone tail by a single bromodomain. *Nature*. **461**, 664–668

9. Patel, K., Solomon, P. D., Walshe, J. L., Low, J. K. K., and Mackay, J. P. (2021) The bromodomains of BET family proteins can recognize diacetylated histone H2A.Z. *Protein Sci.* **30**, 464–476

10. Gamsjaeger, R., Webb, S. R., Lamonica, J. M., Billin, A., Blobel, G. A., and Mackay, J. P. (2011) Structural Basis and Specificity of Acetylated Transcription Factor GATA1 Recognition by BET Family Bromodomain Protein Brd3. *Mol. Cell. Biol.* **31**, 2632–2640

11. Filippakopoulos, P., Picaud, S., Mangos, M., Keates, T., Lambert, J. P., Barsyte-Lovejoy, D., Felletar, I., Volkmer, R., Müller, S., Pawson, T., Gingras, A. C., Arrowsmith, C. H., and Knapp, S. (2012) Histone recognition and large-scale structural analysis of the human bromodomain family. *Cell*. **149**, 214–231

12. Shi, J., Wang, Y., Zeng, L., Wu, Y., Deng, J., Zhang, Q., Lin, Y., Li, J., Kang, T., Tao, M., Rusinova, E., Zhang, G., Wang, C., Zhu, H., Yao, J., Zeng, Y. X., Evers, B. M., Zhou, M. M., and Zhou, B. P. (2014) Disrupting the Interaction of BRD4 with Diacetylated Twist Suppresses Tumorigenesis in Basal-like Breast Cancer. *Cancer Cell*. **25**, 210–225

13. Liu, J., Duan, Z., Guo, W., Zeng, L., Wu, Y., Chen, Y., Tai, F., Wang, Y., Lin, Y., Zhang, Q., He, Y., Deng, J., Stewart, R. L., Wang, C., Lin, P. C., Ghaffari, S., Evers, B. M., Liu, S., Zhou, M. M., Zhou, B. P., and Shi, J. (2018) Targeting the BRD4/FOXO3a/CDK6 axis sensitizes AKT inhibition in luminal breast cancer. *Nat. Commun.* **9**, 1–17

14. Patel, K., Solomon, P. D., Walshe, J. L., Ford, D. J., Wilkinson-White, L., Payne, R. J., Low, J. K. K., and Mackay, J. P. (2021) BET-Family Bromodomains Can Recognize Diacetylated Sequences from Transcription Factors Using a Conserved Mechanism. *Biochemistry*. **60**, 648–662

15. Zou, Z., Huang, B., Wu, X., Zhang, H., Qi, J., Bradner, J., Nair, S., and Chen, L. F. (2014) Brd4 maintains constitutively active NF-κB in cancer cells by binding to acetylated RelA. *Oncogene*. **33**, 2395–2404
